# Spirit of the Foreign Periodicals, &c.

**Published:** 1838-04-01

**Authors:** 


### Spirit of the Foreign Periodicals, &c.

### M. Magendie on certain Auscultatory Phenomena.

The following is an extract from one of M. Magendie's Lectures, recently delivered at the College of France, on the Physical—in contradistinction to the Vital—phenomena of life.

"Another consequence of the existence of elasticity in living bodies is the production of sound.

You know that sound cannot be produced in a body totally inelastic. Thus every species of sound developed in the animal economy is a physical phenomenon, independent of any vital laws, and explicable only on the general laws of physics.

If you are ignorant of this branch of natural philosophy, how will you ever give an account of those numerous varieties or shades of noise, which we hear on exploring certain organs? If you read the magnificent work of Laennec on Auscultation, you will be surprised to find in it so little reference to physical indications and physical laws, although every thing connected with this most important discovery is strictly and truly physical, modified by the conformation and structure of our tissues.

You may, it is true, be able to learn the various *râles*, and to connect them with the lesions whose existence they denote; but, without the assistance of physical laws, you will never have scientific notions on the phenomena of auscultation.

And is there not, in the organization of man, an admirable apparatus destined specially for the production of sound? The organ of voice is *the* instrument of music *par excellence*—an instrument far more perfect than the most finished instrument of art. You hear a man speak, and yet you do not seek to explain by what mechanism the voice is formed! These sounds so varied which issue from the lips, the action of whistling for example, the gurgling noise heard when we gargle the throat, whispering—all these, and a thousand other shades of sound which the human organs are able to produce, are essentially phenomena belonging to the domain of physics.

A surgeon recognises the existence of a fracture by the slight crackling communicated to the fingers by the broken extremities of the bone rubbing against each other. Now what is it that happens on this occasion? The elastic surface of the fragments, rubbed the one against the other, produces certain vibrations; whence results the peculiar sound known by the name of *crepitation*.

Again; the heart in health and in disease gives rise to normal and abnormal sounds, which are explicable only on the ordinary laws of acoustics. You all know the double tictac of this organ: now you cannot conceive the production of this sound without a double shock. But it will be asked, against what does this double shock take place? Oh! it is on this subject that hypotheses and conjectures have been accumulated in such mighty numbers!; and the reason of all this diversity of opinion is to be traced to the single circumstance, that physiologists have rather consulted their own fancies, than studied the laws of natural philosophy.

Hence it is that one set of experimenters has endeavoured to account for the double sound of the heart by the action of the blood against the parietes of the organ, while another set attributes it to the play of the valves, which they compare to the moveable suckers of a pump. *Eh bien!* You will see, at a future part of this course, that, by adapting a sucker to the interior of an elastic tube, and then causing a stream of fluid to be propelled through it, you will in vain try to detect the slightest sound.

Plunge your hand into water, and shake it about with some force, do you hear any noise? Certainly not. And indeed how can it be otherwise? Since the science of physic teaches us, that an essential condition of the shock is the sudden contact of two bodies previously apart. Now if this contact already exists, you cannot expect to have any sound of a shock.

On the contrary, if the heart strikes against the parietes of the chest, (as we shall afterwards demonstrate to be the case by experiment,) this being sonorous, you must necessarily have a sound; for here you have, united, the very conditions which are most favorable to its production.

If now you study those *bruits*, known by the names of the *sawing*, *filing*, and *blowing* sounds, which are occasionally perceptible in the heart and large blood-vessels, their prolonged character or duration will surely not permit you to attribute them to a simple shock, like the tictac of which we have just spoken; and you will find that in truth they are developed under the influence of friction. Natural philosophy instructs us that, when a fluid traverses an elastic tube with rapidity, a sound, such as we allude to, is always audible.

However, we cannot expect to explain the various sounds, which arise from friction, as satisfactorily as those produced by the shock of one solid body against another. We may calculate the force of the latter by knowing the size and density of the two objects, and the strength and quickness with which they meet. Not so with the former.

Experience instructs us what takes place when two ivory balls are struck together; but it informs us very unsatisfactorily of the sounds produced by fluids traversing elastic pipes.

You cannot therefore be surprised that physiologists have not hitherto applied the laws of physical science to these phenomena of the circulatory apparatus; seeing that these laws, even in the case of inanimate bodies, are still unknown, or only very partially so, to philosophers.

The transmissibility, as well as the production of sound, is a phenomenon purely physical, and one which deserves the especial study of the physician. In fact, the transmission of sound through elastic bodies furnishes, in certain cases, the most valuable and positive indications in practice to the medical man. The surgeon, when he wishes to ascertain whether a bone at the bottom of a wound be denuded, or if a calculus exists in the urinary bladder, makes use of a solid instrument for the purposes of exploration; and the simple crepitus or sub-crackling communicated to the fingers by the contact of the sound and the hard body affords the sought-for indication. Vibration is a phenomenon purely physical; for, if instead of a metallic probe or sound, we employ one made of Indian-rubber, the sound produced, as we are well aware, is very different."—*Leçons sur les Phenomenes Physiques de la Vie.*

---

### M. Magendie's Objections to the present Doctrines on the Subject of Contagion.

After examining with great minuteness the various phenomena of imbibition in the living body, this ingenious physiologist directs the attention of his hearers to the absorption of aerial poisons under certain circumstances, or, in other words, to the propagation of morbific miasms from one person to another. His words are as follows:—

"There is a certain number of diseases which are transmissible by the way of contagion. The examination of this subject is very naturally comprised in the study of the phenomena and laws of imbibition.

In truth, we cannot imagine to ourselves any idea of the contagion of a disease, unless we admit the existence of some morbific material, which, proceeding from the body of the sick, is capable of causing a similar train of events in the system of a person in health. Now no substance, whether solid, fluid, or gaseous, can penetrate into the body, except in the way of absorption, however this function is performed. And as contagious diseases are transmitted, not so much by influences at a distance as by direct contact either of the sick or of objects which have been near them, we must apply to physical laws for an explanation of the mode, in which the transmission takes place.

Such is the object which is to occupy our attention at the present moment.

But, before engaging in this disquisition, it may be proper to specify what are the diseases which may be strictly deemed to be contagious; for there are several which have been reported as such, but which certainly are not so.

Look at our Sanitary Code. Is it not a most strange thing to find that these questions, which surely appertain to the department of medical science, have been discussed and settled by men who knew nothing of its principles, and that our medical legislation should rest, as it does, on the most erroneous premises and conjectures? The law recognises five diseases to be contagious, and punishes with death (!) any person who dares to infringe the regulations, which have been adopted to prevent the introduction of any of them into our country. *Eh bien!* out of these *five* diseases, *four* ought to be *erased* from the list. Thus you see what deplorable consequences result from such a state of the existing law.

The whole condition of our (the French) sanitary code, requires a total revision.

We shall now briefly take a glance at these five—reputed to be contagious—diseases, and discuss the principal arguments which are supposed to prove them to be so.

1. *Typhus.*—After the disastrous retreat from Russia, the French army, decimated by a most malignant fever, reached the frontiers of our own country. The dread, which this disease inspired at that time in the people, caused the Government to adopt every measure which, it was supposed, might prevent the introduction of the pestilence.

Medical commissions traversed every department of the country, and issued numerous ordinances; and sanitary cordons were established in various quarters. Notwithstanding all these precautions, the typhus advanced, cutting off great numbers, wherever it broke out, and it soon reached the metropolis. Then our hospitals were so crowded that they were quite insufficient for the number of the military who were attacked.

Everywhere the disease seemed to defy all the precautionary measures, which were adopted to arrest its progress.

Now what conclusion are we to draw from these well-known facts? It is this very important one; that no person would now be so foolish as to propose the formation of any *cordon sanitaire.* In the present day we know better, than we did then, the manner in which typhus is propagated. It is not, as was formerly supposed, by the contact of the clothes, or other coverings which may have touched the body of the sick person that the disease is communicated; but through the medium of the air which is drawn into the lungs in breathing—in short the contagiousness arises from one person inhaling the poisoned or infected atmosphere, exhaled by the other.

Let us suppose a number of persons, labouring under typhus, to be shut up in a close confined chamber, where the air is not readily renewed: you may touch any of them with perfect safety; but if you respire the vitiated atmosphere of the place, charged as it is with the animal particles, which issue from the pulmonary exhalation and cutaneous transpiration, you incur a great risk of being infected with the disease. I have repeatedly seen medical students, on leaving our hospital, where they had gone but once to follow me through the wards, struck with the pestilence, sicken and die in the course of a few days.

Thus I willingly admit the existence of a morbific and contagious principle in typhus, although I deny the manner in which it is supposed to be propagated. From all the researches which I have made, I am now completely convinced that the propagation of the disease is by the medium of the atmosphere and not by the contact of the skin, provided this be not denuded of its epidermis. Remember that, if there be any excoriations on the hand, when you touch a typhoid patient, you may catch the disease by cutaneous absorption, just as easily as you may by pulmonary inhalation.

As to the question of the transmissibility of infectious effluvia by the medium of the atmosphere, we shall return to examine it at greater length when we come to treat of the permeability of the animal tissues by gaseous fluids. At present I have contented myself with merely announcing my opinion as to the genuine character of the contagiousness of typhus fever.

2. *Cholera.*—I have seen and studied this disease in various countries and under all its manifold forms. I have treated more than a thousand patients labouring under it, and have been led to the opinions which I have formed, only after the most patient investigation of the subject. I may fairly say, that as yet I have never met with a single case, which was decidedly communicable, either by direct contact, or through the medium of the respiration. I am aware that many excellent physicians profess the contrary opinion. But I repeat that I have lived, for from eight to ten months, by night and by day, amidst the disease, observing it in all its forms and all its stages; and that the result of all these enquiries has satisfied me that it is never transmitted by *contagion, either mediate or immediate.*

If I were to give my vote as a deputy upon a sanitary code of laws, I should, in all security of conscience, vote that Cholera be expunged from the list of contagious diseases.

3. *Yellow Fever.*—This disease is rarely seen in Europe; but it has been most attentively studied by many accurate observers in many tropical countries, and its history therefore may be said to be well known.

We are assured that yellow fever does not spread by contact from one man to another, but that it is transmitted in the way of infection, in consequence of the impregnation of the atmosphere with putrescent animal particles.

We shall make some experiments on this subject. You will see that a few atoms of certain matters are sufficient to develop in a living body all the principal symptoms, which characterise the yellow fever.

One of the phenomena which we observe most constantly in this disease, especially when it threatens a fatal termination, is the vomiting of black matters. *Eh bien!* If you introduce into the sanguiferous system of a dog a few drops of water, which has rested in contact with some putrid fish or flesh, you will find the animal exhibit a singular restless activity: fever is quickly kindled up, the animal lies down, refuses food, and begins to vomit enormous quantities of those black matters, which constitute so characteristic a symptom of yellow fever.

We know also that, in all the circumstances, under which yellow fever is developed, the air has been vitiated by the disengagement of putrid animal effluvia. Thus it is not uncommon to see it break out when a vessel, loaded with codfish (morue) has stranded in the neighbourhood of a village, and the cargo has been left to putrify on the beach. If the putrid mass be thrown into the sea, the fever soon ceases. Thus, when the cause which engenders and keeps up the yellow fever is perfectly well known, we only require to protect ourselves from it, to lose all dread of this terrible pestilence. It is very different with the Cholera; for as yet we are utterly ignorant of the circumstances which favour its development.

What however, I have said of the Cholera, in reference to its alleged contagiousness, is equally applicable to the Yellow fever: it ought to be erased from the list of contagious diseases.

4. *Lepra.*—To witness the reception which a leprous patient undergoes upon his arrival at an hospital, the care with which he is examined, the anxiety of the physician to have a drawing taken of his appearance, and the numerous visitors who come out of curiosity to look at him, we should certainly never dream of the rigour of the law, which makes it a capital offence to have communicated with a person who is altogether so interesting!

Lepra is one of the five contagious diseases enumerated in our Sanitary Code.

The good sense however of the public has done justice, where the legislature has been ridiculous and cruel. The folly of the enactment still subsists in the statute book.

As to the lepra which prevails in hot climates, it seems that the unfortunates, who are afflicted with it, are separated from the rest of the community, and that the dread of contagion is still very general. As I have not seen it myself in these climates, I have not been able to form a decided opinion upon the subject, although I am strongly induced to believe that authors have very much exaggerated the arguments, by which they have endeavoured to prove the contagiousness of this disease.

5. *The Plague.*—Of all the diseases, reputed to be transmissible, this one demands the most attentive and conscientious examination from us; for its destructive ravages fill every one with alarm and apprehension. If, as is affirmed, it can be conveyed by linen, cotton, and woollen substances, by the skins of animals, and by numerous other articles enumerated in our sanitary regulations, we should be among the foremost to applaud the strictness of our quarantine laws. But examined in a medical and scientific point of view, is it really the case that the contagiousness of the plague has been satisfactorily proved? It is by a tradition which may certainly be traced back for many ages, that this disease has been considered to be transmissible in the way of contagion. Perhaps therefore, the opinion is quite as much attributable to the dread which the disease has universally inspired, as to any absolutely correct data on the subject. Whenever the plague has broken out in any place, a general dread has usually seized all the inhabitants round about; every one hurries to fly off, and if any one is so rash as to hold any communication with an infected person, the rest of the people have refused to admit him among them. Thus, fear alone has pronounced upon the character of the disease; and fear, as you well know, has not a very accurate judgment.

I have myself visited most of our towns which are provided with lazarettoes, and have had much intercourse with the physicians settled in these places; and I have found several of these gentlemen very sceptical as to the contagiousness of the plague. They are however prudently reserved in giving a decided opinion to this effect, as such conduct would do a serious injury to their practice. If a physician at Marseilles or Toulon were so bold as to question the contagiousness of this malady, he would inevitably expose himself to general reprobation—so universal is the dread of its importation. Let us for a few moments cast a glance over the sanitary precautions adopted in French and other Lazarettoes.

The fundamental idea, on which rests the medical police of these establishments, is that the plague is propagated by contact, and by contact only; and that it is never communicated from one individual to another through the medium of the atmosphere. You are permitted to enter into the chamber of an infected person, provided you are first enveloped in a "assez bizarre" dress. You are covered with a large *domino* of gummed taffetas, and the hands are provided with gloves of the same stuff. With such an apparatus it is believed that you can incur no risk in approaching the beds of the sick. As long as you avoid direct contact with them, or with the clothes which they wear, you are supposed to be quite safe; but if you happen to touch either with your bare finger, oh! then you are thought to be inevitably infected.

Such is the basis of the existing doctrines upon the contagiousness of the plague; and every arrangement in our lazarettoes is founded upon it.

The regulations respecting the purification of suspected goods, such as bales of wool, cotton, &c., are equally ridiculous. How, for example, do you suppose that the quarantine officers proceed when they wish to determine whether a bale of goods contains the germ of the plague? The proof, to which it is subjected, is as follows.

A porter plunges his arm into the midst of the suspected bag, shakes it about in all directions, then withdraws it, and closes the bag. Such is the manœuvre which I have myself repeatedly witnessed. If the man does not exhibit the symptoms of the plague in the course of a fortnight after the experiment, it is inferred that the goods are not infected!

Now I ask of you, can any proceeding be more ridiculous? How! because a man may plunge his arm into a bag of cotton, and yet escape unhurt, are we to infer that it is entirely free from any infectious miasms.

Besides; why should we refuse our belief in the transmissibility of the contagion of the plague through the medium of the atmosphere, when we know for certain that other epidemic diseases, such as variola, rubeola, &c. are often propagated in this manner?

Remember; that we do not at all call in question the fact that woollen or cotton goods, &c. may hold, and retain, infectious particles. All that we object to is the strange doctrine, that these particles should not be disseminated through the atmosphere and in this state exert their ordinary influences.

That the mode of testing the purity or impurity of goods—we mean whether they be infected or not—is, we need scarcely say, ridiculously defective. It is indeed, surprising, that such antiquated practices should subsist in France, so enlightened as our countrymen are upon most other subjects of medical police and jurisprudence.

If the skin covered with its epidermis has such extraordinary powers of absorbing the noxious particles, how comes it that the tender and delicate surface of the mucous lining of the air-tubes should be so impenetrable? We cannot find any rational interpretation of this anomaly; and we are therefore obliged to question the accuracy of the statement. As long as the epidermis remains uninjured, it is scarcely possible to suppose that the absorption of any morbific particles can take place by it. *It is much more probable to imagine absorption by the pulmonary than by the cutaneous surface.*

Were I to enumerate to you all the rules and regulations of the lazaretto at Marseilles, and to mention all the ridiculous usages practised there, you would be utterly astonished that, in the year 1837, and in a country like ours—where all the physical sciences are cultivated with such extraordinary success, and have shed such a lustre over every department of knowledge—our commerce, our navy, and our travellers, should still be subjected to the most absurd and useless impediments."

M. Magendie continued the discussion of this interesting and most important subject at his following lecture.

"You have heard," said he, "that our government recognises *five* diseases to be essentially contagious, and that *four* of these, viz. typhus, cholera, the yellow fever, and lepra, are in truth not contagious at all. As to the fifth one, the plague, I am unable to come to a decided opinion on the subject, until I am provided with more numerous and authentic data. The mere supposition that the contagious miasm or principle is communicated by cutaneous imbibition, and not by pulmonary absorption, is a paradox contradicted by all sound physiology. As therefore this opinion does not rest upon the results of any direct experiments, and is still enveloped in a cloud of prejudices, rendered still more dense by fear, it is quite necessary that the subject of contagion be examined with much greater attention than of late it has been.

Can we require a stronger proof of such necessity than the circumstance that M. Clot Bey, the distinguished surgeon of the Pacha of Egypt, denies that even the plague itself is communicable by mere contact. M. Brayer, too, an eminent physician, who has lately returned from a long residence in Constantinople, is of opinion that it is not transmissible from a sick to a healthy person, (ne peut se transmittre de l' homme malade á l' homme sain.)

The very rigour of our quarantine laws must prevent their being duly enforced. Call to mind what happened in Paris during the late epidemic of the cholera. On my return from a journey to the north of England, whither I had gone for the purpose of studying this new pestilence, I passed through Boulogne. Finding myself one evening with several intelligent inhabitants, I was told that a foreign vessel, which had attempted to enter the port before she was visited by the health officers, had been fired upon from the fort. I said to them, 'Gentlemen, if any one has brought cholera amongst you, it must be myself; for I have this very day come from the places where it is raging with great violence, and the clothes which I am wearing at present are the same which I had on when I was visiting the sick. So you see that we are all compromised for the quarantine.' As every one present had a personal interest to keep the affair secret, the news were never told. My friends however, cautioned me to be more discreet when I got to Paris; for otherwise, most assuredly, I should be shut up in a lazaretto! *

With respect to certain diseases, such as small-pox, hydrophobia, syphilis, &c. there cannot be a doubt that they are communicable in the way of contagion. The physical conditions of their transmission are well known; but the special agent, which serves to re-produce the morbid action, has hitherto quite escaped our most subtle enquiries. How, for example, does the purulent matter, secreted from a syphilitic sore, when it is deposited on any surface from which it may be absorbed, give rise to a reiteration of the same phenomena in the infected person?

No chemical discovery has ever been able to point out the difference between common—or, what used to be called, laudable—pus, and the discharge from a venereal ulcer. Various contrivances have been proposed to guard against contamination in impure connexion. There is a powder which is sold in the chemist's shop for this purpose. It consists partly of chalk; and is to be sprinkled on the glans penis before coition. Other people recommend the use of lotions of various kinds, such as of eau de Cologne, a solution of the chlorurets, or of corrosive sublimate, or of liquor potassæ, or of nitrate of silver, &c.

All these substances may probably act in the same way; viz. by modifying, to a certain degree, the state of the epidermoid covering of the glans and prepuce, and thus rendering it less greedy of absorption. But they are far from giving security against infection, and "le moyen le plus sur de prevenir l'infection est encore de s'abstenir."

The Itch is another disease which is readily communicable by contact. In the case however of this disease, it is not a morbid virus that propagates the mischief, but an insect, which may be discovered with the aid of a magnifying glass.

As to the small-pox, measles, scarlatina, &c., the mechanism of their transmission is doubtless through the medium of the atmosphere and the absorbents of the lungs. It is therefore unnecessary to dwell more minutely upon their history."—*Leçons sur les Phenomenes Physique de la Vie.*

* We need scarcely remind our readers that the regulations, adopted in this country to prevent the importation and dissemination of the cholera, were as ridiculous as those of the French government.

While an uninterrupted communication by land existed between an infected place and all the other parts of the island, there were strict prohibitory restrictions imposed on vessels coming from it, if it happened to be on the coast, to other sea-ports!—*Rev.*

---

### M. Magendie on some of the Phenomena of Absorption.

"The act of the absorption of any poisonous matter is composed of two distinct periods, imbibition in the first place, and secondly the passing of transportation of the imbibed matter.

In the present day no physiologist doubts that the venous system is the apparatus of absorption. This is a fact simple and at the same time so palpable that we cannot hesitate for a moment to question its truth.*

* The bold assurance of M. Magendie in many of his assertions is somewhat staggering at first to the cautious enquirer after truth. In course of time, as we become better acquainted with his writings, we are rather amused than angry with him for his style.—*Rev.*

If, instead of experimenting upon the minuter vessels, we study the phenomena of absorption on the larger blood-vessels, we can readily trace all the phases or successive stages of the process; we can see the absorbed substance pass through the parietes of the veins, follow the current of its blood, and then be immediately conveyed towards the great centres of the nervous system.

I will perform such an experiment before your eyes, in order that you may be completely satisfied of the truth of what I now tell you.

I lay bare the jugular vein of a dog, and, having exposed it for some extent, I separate it from the subjacent tissues by placing a small portion of card under it. Thus insulated, the vessel communicates only with the capillary vessels superiorly, and with the central organ of circulation inferiorly. I now place a few drops of tincture of nux vomica—which has been previously warmed, for the purpose of favouring the imbibition—on the coats of the vein. The subjacent card prevents any of the tincture falling upon the surrounding tissues of the part; and all chance of absorption by them is thus prevented.

You observe that the effects of the poison are very slow in being manifested; for already five minutes have passed, and the animal does not exhibit any sign of inconvenience.

This tardiness of action is just what we might have anticipated; since the poisonous matter is in contact with one vein only, and not with numerous capillaries, as in the ordinary application of poisons to the body. But now some of the symptoms of poisoning begin to declare themselves: Eh bien! I can at once stop them by tying the two extremities of the vein. You see this; the animal becomes quite calm in a moment. Here then you have an indisputable proof of the absorption by the veins.

Let us now examine the state of the vein, on which we have just experimented. Its parietes have lost their natural colour, and assumed that of the substance which has penetrated their tissue.

If you touch with the point of your finger the internal surface of the vein, and then apply it to your lips, you will at once recognize the bitter taste of the Nux vomica.

There must, therefore, have been a transmission of the substance from the outside to the inside of the vessel. The poisoning has in this experiment been produced, in the same manner, as when we inject directly any deleterious matter into the sanguiferous system: in the one case the matter has penetrated through the natural porosities of the vessel, while in the other it has been introduced by a puncture in its parietes.

I speak not at present of the lymphatic vessels; for you are aware that they are not permeated, like the veins, by regular currents, and their part in the act of absorption must be next to nothing, (leur rôle dans l'absorption doit etre à peu près nul).

The following is a curious fact, which I have observed more than once. In the horse, on which I performed the experiment of injecting 30 *litres* of air, we found, on the dissection of the body, the lymphatic system enormously distended with lymph. It would thus seem that the considerable pressure, which the injected air exerted on the blood in the blood-vessels, extended even to the contents of the lymphatics, and thus caused the distention which we have mentioned.

To complete our examination of the important function of absorption, let us, for a few moments, consider some pathological phenomena which are connected with it, and which may justly be deemed *ready-made experiments* on man.

You are aware of the œdematous infiltration which are apt to take place, whenever the circulation is impeded; whether this disease proceeds from a deficient energy in the heart itself, or from some mechanical obstacle to the free return of the venous blood.

Every theory of dropsy, be it general or merely local, rests on the great phenomenon of venous absorption.

We are indebted to M. Bouillaud for several applications, in practice, of the pathology of these physiological facts.

When the inferior extremities only are œdematous, you will usually find that the obstacle to the return of the venous blood exists in the crural vein: in some cases, its cavity is occupied with fibrinous coagula, while in others, it is pressed upon by an adjacent tumor. What happens in such cases? The blood is more or less retarded, or even stagnates altogether from the point, where the obstruction is situated, to the minute capillaries; and, as the arterial exhalation continues as before, the absorption by the veins can no longer take place: hence there results an accumulation of serosity in the meshes of the cellular tissue. It is thus that, in cases of obliteration of the vena portæ, a dropsical effusion into the cavity of the abdomen is induced.

Of late years, some authors have treated of what they term Ascites of the brain. But in truth they have mistaken, under this appellation, the natural secretion of the cerebro-spinal fluid for a pathological lesion.

The genuine Œdema of the brain consists in an accumulation of serosity in the very parenchyma, so to speak, of the nervous pulp, or in the cavities of the cerebral ventricles. We may readily imagine that any impediment to the reflux of the blood from the head towards the heart, such as concretions in the sinuses of the dura mater, &c., may give rise to this cerebral dropsy."—*Leçons sur les Phenomenes Physiques de la Vie.*

---

### Experiments on the Spermatic Animalculæ, and on some of the Causes of Sterility in Women.

*Importance of the Use of the Microscope in Physiological Enquiries.*

M. Donné, whose name we have had occasion of late years to mention with praise as an able and ardent investigator of physiology, is the author of the following observations, communicated to the Royal Academy of Sciences at Paris.

The spermatic animalcules (*zoospermes*) have been submitted to numerous examinations since the days of Lewenhœck, who was the first to describe them with any degree of accuracy; but hitherto little progress has been made in the discovery of their natural history, or of the part which they perform in fecundation.

M. Donné has studied them in a new point of view. He placed them in contact with the principal fluids of the body, for the purpose of watching the effects of these fluids on their vitality, and general phenomena.

By following out this train of enquiry, he has been led to conjecture that certain changes in the properties of the mucus of the vagina and of the uterus may exercise a deleterious influence on the animalculæ of the male semen, and may thus as effectually prevent impregnation, as if there was a radical defect in the female organs of generation.

In this point of view, we perceive that some light may possibly be thrown on the hitherto most obscure subject of *sterility*.

M. Donné commenced his researches by examining the effects of the blood itself, of milk, of the healthy vaginal and uterine mucus, of the purulent discharge in syphilis and in gonorrhœa, of the saliva, and of the urine on the spermatic animalculæ. He found that they continued to move and live in some of these fluids; while in others they immediately perished.

Thus blood, milk, and pus did not seem to have any visible effect upon them; but the urine and the saliva appeared to kill them at once. The mucus of the vagina and of the uterus was, as might be expected, perfectly innocuous; even the presence in it of new infusoria—which have been discovered by M. Donné in certain vaginal discharges, and to which he has given the name of *trico-monas*—did not appear to affect the spermatic animalculæ.

There are some cases however, in which the vaginal and uterine mucus has a noxious influence. The investigation of this point constitutes the most important theme in M. Donné's memoir. He has found, we are told, that the mucus of the vagina in some women, apparently in perfect health, is such that the spermatic animalculæ perish immediately in it.

This noxious quality belongs sometimes to the vaginal, at other times to the uterine mucus. Having satisfied himself of this fact, he next enquired whether the mucus exhibited any appreciable changes of quality from its normal properties and condition; and he thinks that he has succeeded in discovering certain traces of such changes in the chemical constitution of the secretion.

He says that the mucus of the vagina, from the vulva to the os tincæ, is always *acid*, whereas that of the cervix and body of the uterus is always *alkaline*. (!) Now he supposes that in certain habits and in certain states of the system, there is a disposition to excess of acidity in the one fluid, and to an excess of alkalinity in the other. The probability of these novel ideas rests upon the results of several experiments, which M. Donné has lately performed.

The second part of M. Donné's Memoir is occupied with the investigation of involuntary seminal discharges. This subject has been recently examined by M. Lallemand of Montpelier; but his work is rather of a practical than of a physiological nature.

The chief object of our author's researches has been to discover, if possible, some sure diagnostic signs, by which the presence of seminal matter may be recognized in the urine. He has very satisfactorily shewn that the signs, which the Montpelier professor has pointed out for this purpose, are quite nugatory. The chief of these are the thick and troubled state of the urine, its sickening fetid odour, a cloudy flakiness through it, and a glairy filamentous and greenish-coloured deposit adhering to the bottom of the vessel.

Now all these characters may exist in urine, which has no admixture of seminal fluid; and, on the contrary, spermatic animalculæ have been detected by the microscope in urine, which was quite limpid and transparent, or perhaps only slightly mucous.

It is by the use of the microscope alone that we can hope to discover the presence of the seminal fluid; and this mode of diagnosis is the more satisfactory, as it seems that the characteristic form of its animalculæ is not at all altered by the action of the urine. As their specific gravity is greater than that of the urine, they always fall to the bottom of the vessel; and thus the smallest quantity of semen is readily discovered.

To remove all source of ambiguity, M. Donné has made several experiments to ascertain whether there is ever semen present in the urine, when the person is in perfect health, and has no symptom whatsoever of seminal weakness.

He thinks that he has quite satisfied himself that such is never the case in health, unless indeed there has been an emission shortly before the urine was discharged; for under these circumstances there are always some animalculæ discoverable, according to his researches. With the exception of such a case, he has never detected them in the urine of a person in health; and he therefore points out the great importance of attending to this symptom—the existence of spermatic animalculæ in the ordinary urine—before the disease has made much progress.

The memoir concludes with the reports of several cases, in which the existence of seminal weakness was suspected, and where M. Donné was requested to examine the urine with the microscope. In some, the presence of the spermatic animalculæ was readily discoverable; while in others the suspicion was proved to be groundless.

In closing these brief remarks, we cannot too urgently impress on the attention of medical men the very high importance of using the microscope more frequently, than they have done hitherto, in examining the products, healthy as well as morbid, of animal life. In conjunction with the valuable assistance of chemistry, this simple instrument promises to reveal many of the mysteries of life and of its wonder-working operations.—*Gazette Medicale de Paris.*

### Case, where the Fœtus was heard to cry before birth.

The occasional occurrence of the fœtus being heard to cry during labour, while it is still within the uterus, is placed beyond doubt by numerous cases recorded by most unexceptionable authorities.

The following may be added to the number. A woman was in labour with her fifth child; the membranes had broke, and a large quantity of the waters had been discharged. Shortly afterwards the fœtus was heard to cry distinctly five or six times, not only by the nurse, but also by the patient herself and her husband.

At this time the forehead of the child was found to be resting on the brim of the pelvis, and its face could be easily felt with the finger. The extraction of the head was effected by means of the forceps; but not without some difficulty. The child did not breathe at first; but was re-animated by the use of the ordinary means.

*Remarks.* It will be observed that, in the preceding case, not only had the membranes been already ruptured, but also that the face of the child was directed towards the orifice of the uterus at the time when it was heard to cry. The woman was sitting—as is the custom on the Continent—on the *chaise d'accouchement*.

Some writers have asserted that a distinct *vagissement uterin* has been heard, even before the membranes have given way. This opinion however is more than doubtful.

M. Marc, in his able Memoir on Infanticide, in the 2nd edition of the Dict. de Medecine, observes:—"In all the cases of uterine crying, while the head has not yet reached the orifice of the vulva, there has been the discharge of the waters some time previously; and in most of the cases frequent manual attempts have been made by the accoucheur to effect the delivery."

The possibility of the child respiring before its delivery is of high importance to the medical jurist; as it shews that the mere circumstance of the lungs containing atmospheric air cannot be received as a sufficient proof that the child has been born alive.—*Bulletin Medicale Belge.*

---

### Remarks on Insanity—important Distinction between two of its Forms.

M. Leuret, long a pupil of the famous M. Esquirol, and now one of the physicians of the Bicetre Hospital at Paris, commences a very valuable contribution on insanity to the Gazette Medicale by pointing out the important difference which he wishes to establish between *hallucinations* and *lunatic* or *delirious ideas* or *conceptions.*

The former term he proposes to limit to those aberrations of the mind which are always *connected or attended with* some physical or corporeal uneasiness, pain, or any other distressing feeling in a part. For example, it is not unfrequent for a madman to believe that there is a devil lodged in his bowels, or that his head is made of glass, or that his hand is made of ice, and so forth.

These are instances of genuine *hallucination.* The morbid train of ideas springs from, or at least is associated with, an uneasy sensation in some part of the body: for example, it is pain in the bowels which causes the madman to believe that a devil has taken up his abode there; it is the feeling of cold numbness that makes him suppose that his head is made of glass, or that his hand is made of ice, and so forth.

Many of the vagaries of the mind during dreams are explicable on the same principle.

A bodily feeling gives rise to a train of ideas, which the mind, uncontrolled either by perception or by judgment, follows out oftentimes in the most curiously discursive manner.

We dream perhaps that we are climbing Mont Blanc, or Ætna, and that we are treading over the eternal snows of the one, or over the heated lava and ashes of the other—the "point de depart" having consisted in the simple occurrence of our feet, while we are asleep, having been either uncovered by the bed-clothes in a frosty night, or perhaps resting on a bottle of hot water. Again, how often does the debauchee's dream turn upon burning deserts, or the sting of serpents causing intolerable thirst, or on the infernal regions themselves—the parched state of his mouth having given rise to this train of ideas!

Such then is the true nature and character of *hallucinations*, according to the language of M. Leuret.

On the other hand, *delirious ideas* or *conceptions* (idées ou conceptions delirantes) are those visionary and insane creations of the mind, which seem to spring up quite independently of any corporeal sensations; they are the pure fabric of the fancy, and have no reference to any one part of the body.

For example, a youth, under the care of M. Leuret, imagined himself to be the son of Napoleon, and heir of all his greatness. He looked down upon all his family as utterly unworthy of his attention, and regarded himself as one of the kings of the earth.

Another man, who had been a baker, and had once served in a regiment of infantry, imagined that he had become one of the Marechals of France; a third, that he was a special messenger from heaven, to reveal the will of the Almighty.

It will be observed that in none of these three cases, can any reference be made to a mere bodily sensation, as the cause of the mental aberration; it was a purely mental delirium, a mere phantasm of the imagination.

Having in this manner pointed out the difference between *hallucinations* and *delirious conceptions*, M. Leuret proceeds to remark that the treatment of these two forms of insanity must be conducted on different principles. The one invariably requires a physical or corporeal, as well as a mental regimen. Whereas for the cure of the other we must trust chiefly to the mental or moral treatment.

Thus in the case of the youth who fancied himself the son of Napoleon, M. Leuret speedily effected a cure, and checked all his vagaries by exacting from him an entire submission to his orders. The hero-born youth at first attempted to resist the command of a mere doctor. A few cold baths however speedily disabused him of all his fancied greatness, and in the course of a month or two he was comparatively sane.

Again in the case of the baker, who had mounted up the ladder of vanity, till he became a Marechal of France, M. Leuret acted in the following manner.

He induced his patient to give him a short history of the events of his youth. The man was flattered by the attention of his doctor, and he told him every thing about his father and mother, his associates, the bake-house, &c.

As he approached the period of his life from which his insanity might be said to commence, M. Leuret, under the guise of curiosity, requested him to repeat once more his early history; in order to cause his mind to dwell very forcibly on the field of reality. Hitherto his language and behaviour had been calm and modest. But as he began to describe his rise in the army through the successive ranks of lieutenant, captain, up to those of general and marechal, he became very serious and lofty. M. Leuret, suddenly interrupting him, said very angrily: "think you that I am in a humour to listen to such nonsense? You know that you are only a baker's man; for you have this moment told me so. I had intended at first to have done you a kindness; but after such abominable falsehoods you have lost all my good will. Leave the room immediately."

He was very unwilling to go, and besought M. Leuret to listen to him; but the doctor, purposely designing to convince him of his folly, refused to see him for some days.

When M. Leuret next summoned him to come before him, he refused, and he was therefore forced into the room. He was sulky, and at first would not answer. M. Leuret told him that he was very unwilling to subject him to the cold douche bath and other indignities, which he reserved only for rogues and liars, but that if he persisted in his assertions, he should most certainly be treated like the others. The use of the cold baths was very galling to him; so M. Leuret once more tried to dissipate his extravagancies by reasoning with him.

This time he succeeded; the man was induced to go into the bake-house of the hospital, and there made himself useful as a clever workman. His state gradually improved, and when he was discharged, his mind had nearly lost all its propensity to aberration.

The preceding may be regarded as a genuine case of a purely mental disturbance, unconnected with any corporeal indisposition; in short of what M. Leuret calls '*une idée delirante*' in opposition to '*une hallucination*'—which, as we have already explained, proceeds from, or, at least, is associated with, a painful or uneasy sensation of some part of the body.

Moral treatment, combined as a matter of course with due attention to the general health of the system, will generally succeed in dissipating the illusion in the former set of cases. It is a great mistake, says M. Leuret, for the attendants of such lunatics to give in to their extravagancies: such concession is only confirming the delusion in their minds.

In the treatment of '*hallucinations,*' it is quite necessary to have recourse to the relief of the existing morbid sensation at the same time that we attempt the removal, if possible, of the mental delusion.

For example; a man suffered from headache, and believed that there were two horns growing from the crown of his head. Nothing could disabuse him of this strange notion. It was therefore proposed to remove the appendages by a surgical operation. A deep incision was made through the scalp and the surgeon made a shew of sawing off the excrescences. When the operation was completed, two ram-horns, which the surgeon had brought in his pocket, were shown to the patient, who readily believed that they had been removed from his own head; and he ever afterwards was satisfied.

Pinel mentions the case of a woman, who also suffered from severe head-ache, and who imagined that a worm had got into her brain and was gnawing it away. It was proposed to remove it by an operation. The scalp was divided, and a long clot of blood was drawn out from the wound, and shown to the patient as the cause of all her sufferings. Her mind was at once relieved from her former delusion.

In the treatment of these two cases the combination of physical and moral treatment was eminently successful. They were both genuine examples of *hallucination.—Gazette Medicale.*

---

### Increase of Insanity with the Progress of Civilisation.

M. Brierre de Boismont, at a recent *seance* of the Royal Academy of Paris, communicated the results of certain enquiries which he has of late made respecting the number, actual as well as relative or proportional, of insane persons in the different leading towns of Europe.

From these it appears that the melancholy reflection, that insanity increases in frequency progressively with the advance of civilisation and refinement, is but too true. While London and Paris exhibit an appalling catalogue of hundreds and thousands of lunatics, Madrid and Cairo shew comparatively but very few. The feverish play of passion, the hot thirst of ambition, the sting of disappointment and chagrin, the blighted hopes, the fears and cares and sorrows of man in a highly artificial state of society—all these 'vultures of the mind' keep pecking at its fine network, until it becomes a tangled and confused web. In the number of the Annales d'Hygiene for January 1835, it is justly remarked: "The occupations, amusements, follies, and above all the vices, of the present age are infinitely more favourable to the development of insanity than at any previous period.

We live under the dominion of the *propensities*, and must pay the penalty for so doing: madness is one of these.

More cases owe their origin to moral than to physical causes. If we consult M. Esquirol's table, published in 1835, comprehending 1557 cases, and exclude 337 instances of hereditary taint, it will appear that 579 are attributable to the excess or abuse of the passions, or to the weakness of the uneducated intellect." The following table affords a sad confirmation of the truth of these remarks.

| Cities. | Population. | No. of Insane. | Proportion. |
|---|---|---|---|
| London | 1,400,000 | 7,000 | 200 |
| Paris | 890,000 | 4,000 | 222 |
| Petersburg | 377,000 | 120 | 3,133 |
| Naples | 370,000 | 479 | 759 |
| Cairo | 330,000 | 14 | 30,714 |
| Madrid | 204,000 | 60 | 3,350 |
| Rome | 154,000 | 320 | 481 |
| Milan | 151,000 | 618 | 242 |
| Turin | 114,000 | 331 | 344 |
| Florence | 80,000 | 236 | 338 |
| Dresden | 70,000 | 150 | 466 |

True it may be that many of these numbers are far from being exact, or from exhibiting a correct view of the actual amount of insanity in some of the cities mentioned.

In such as Cairo, Madrid, and Petersburg, there may possibly be a great number of cases, of which the enquirer can obtain no information, in consequence of the want of proper asylums, and accurate registers.

The statistics of insanity in many countries are so imperfect, that we can place but little reliance as to the exactitude of their results. Still however the general conclusion cannot be gainsayed that the relative number of the insane to the actual number of the inhabitants, of the large towns in the above table, is very nearly in exact proportion to their civilization and refinement.

The following table given by M. de Boismont confirms but too truly this assertion.

| | Population. | No. of Insane. | Proportion. |
|---|---|---|---|
| *State of New York | 1,617,500 | 2,240 | 721 |
| England | 12,700,000 | 16,222 | 783 |
| Scotland | 2,093,500 | 3,652 | 563 |
| Norway | 1,051,300 | 1,909 | 551 |
| France | 32,000,000 | 32,000 | 1,000 |
| Belgium | 3,816,000 | 3,763 | 1,014 |
| Holland | 2,302,000 | 2,300 | 1,046 |
| Italy | 16,789,000 | 1,441 | 4,879 |
| Spain | 4,085,000 | 569 | 7,181 |

ON THE DURATION OF LIFE IN FRANCE SINCE THE COMMENCEMENT OF THE PRESENT CENTURY, BY M. BIENAYMÉ, INSPECTOR OF FINANCES, &c.

*(Read before the Institute.)*

In noticing this elaborate paper of M. Bienaymé, we cannot enter upon the minute discussions, with which it is chiefly occupied. All that we propose to do is, to present to our readers some of the most instructive tables, or registers, which are contained in it.

The following one exhibits, in round numbers, the relative numbers of male and female births in France from the year 1817 to 1832 inclusive:—

| *Years.* | *Boys.* | *Girls.* |
|---|---|---|
| 1817 — | 488,000 — | 455,000 |
| 18 — | 471,000 — | 442,000 |
| 19 — | 509,000 — | 478,000 |
| 20 — | 494,000 — | 464,000 |

* The following passage is taken from a recent work, entitled, 'What Asylums were, are, and ought to be,' by W. Browne, surgeon of the Montrose Asylum.—"The number of lunatics is said to be much greater in America than in any European country. Can this be the effect, it has been asked, of the acquisition of independence, or of the operation of the constitution under which the people live? I am disposed to believe that a concurrence of causes may have produced this result. First, the abuse of ardent spirits.... Secondly, money is gained easily and rapidly, and the abject and ignorant become suddenly rich, without becoming wiser or better.... Thirdly, without wishing to repeat the heartless sneer that the Adam and Eve, of the United States were born in Newgate, the fact cannot be overlooked that the sources of the tide of population, which has been flowing uninterruptedly towards America, have been impure and poisoned.... Fourthly, the intenseness of political feeling, and the agitating nature of the civil contests must decidedly contribute to the development of the disease." *Rev.*

| | | |
|---|---|---|
| 1821 | — 497,000 | — 465,000 |
| 22 | — 501,000 | — 471,000 |
| 23 | — 496,000 | — 467,000 |
| 24 | — 507,000 | — 476,000 |
| 25 | — 503,000 | — 470,000 |
| 26 | — 511,000 | — 481,000 |
| 27 | — 505,000 | — 474,000 |
| 28 | — 501,000 | — 474,000 |
| 29 | — 496,000 | — 468,000 |
| 30 | — 496,000 | — 470,000 |
| 31 | — 509,000 | — 477,000 |
| 32 | — 483,000 | — 454,000 |

The author afterwards gives another table shewing the entire number of births and deaths in France from 1803 to 1811.

The total amount is 8,265,950 births.

and 7,279,387 deaths.

The average yearly } 918,440 births.

may be stated at } 808,861 deaths.

If we now divide the annual number of births, according to the average proportion deducible from the preceding table, into male and female births, we find that of the 918,440 children born each year, 475,880 are boys, and 442,550 are girls.

From an extended series of calculations, M. Bienaymé has ascertained that, in the present day, of every 100 male births, rather more than 60 survive to the age of 20—the age when they are liable to serve as recruits in the army. The number of female children who live to this age *must be still greater* (doit être plus grande encore); for it is a fact, established beyond all doubt, that the female sex possesses at almost every age, even previously to child-bearing, the privilege of dying in a less proportion than the male sex.* (The translation of this sentence is designedly quite literal.)—*Annales d'Hygiene Publique, Aout* 1837.

---

*This curious fact—the less relative mortality among females than among males,—ought to be known to medical men, who, we regret to state, do not take sufficient interest in the study of statistics.

### Report on the Evils of the Factory System in France.

*(Read before the Institute.)*

MM. Villermé and Chateauneuf were recently appointed by the Academy of Moral and Political Sciences to examine and ascertain, in the different departments of the kingdom, "the moral and the physical state of the working classes."

These two gentlemen took different directions of the country for inspection. While the latter travelled through the central districts and along the sea-coast, M. Villermé (who is the reporter on the present occasion) visited those departments in which there is the greatest number of cotton, woollen, and silk manufactories.

He states, that there is at all times a large number of the manufacturing population in France in real misery. With scarcely sufficient clothing during the day, and ill-sheltered at night, their food is but too often scanty and of the coarsest description. The hours of labour are in many cases far too long. Each day both males and females are often occupied from fifteen to fifteen and a half hours; and of these fully thirteen are devoted to actual hard work. The condition of the children in these manufactories is often still more deplorable than that of the adults; their hours of work being quite as long, while their strength is less able to bear it.

In the province of Alsace, many of these youthful unfortunates are children of Swiss or German families, which have been reduced by distress. To add to their other calamities, the price of lodgings in the manufacturing towns is often so high, that they are obliged to live at some distance.

"No where," says M. Villermé, "is the wretchedness more conspicuous than at Malhouse, a town which, although it has greatly increased in size of late years, cannot contain all its work-people. Every morning we see hundreds of miserable, half-fed, ragged children, arriving from all the environs and hurrying, perhaps, through the rain and mud with their morsel of bread, which is to suffice for food till their return home."

The children, employed in the cotton manufactories in the department of the Haut-Rhin, are somewhat better cared for; but even there, the contrast of the factory children with those in the adjoining rural districts, is very grievous. Instead of the blooming health and gay spirits of the latter, they are almost all pale, weak and dispirited looking, ill-clad, ill-fed, and altogether wretched. How can we be surprised at this, when we call to mind the hours of labour to which they are doomed? They are actually, in many cases, longer and more severe than what the galley slaves have to endure. Twelve hours—and of these two at least are allowed for food—are the most that are ever exacted from these; whereas the children in very many manufactories are, as already mentioned, condemned to fourteen or fifteen hours, out of the twenty-four. The age of these unhappy children is usually from seven to thirteen years.

M. Villermé mentions that the children, employed in the woollen manufactories, are on the whole better off, than those in the cotton ones. The workshops are more healthy, the wages are somewhat higher, and the children employed in the former are usually two or three years older, than those engaged in the latter.

It is true that no hard manual work is imposed on the children in either case—their duty consisting chiefly in 'une simple surveillance.' But then to require them to stand in one confined spot hour after hour, without change of place or even of attitude, cannot but be pernicious to their health in the extreme. And after their long day's work is over, to be obliged to walk two or three miles home to a scanty supper and a damp unwholesome bed—what a hard and cruel fate! Let us not hear of the miseries of West India slavery: the lives of our own fellow-countrymen is worse a hundred fold.

M. Villermé calls upon his countrymen to follow the generous example, which has been set by the government of Great Britain, in restricting the number of hours of work to which factory children should be subjected. He alludes to one or two most laudable efforts which have been made by the manufacturers themselves to introduce a better state of things; and addresses from several of the commercial towns throughout the kingdom have been addressed of late years to the minister of commerce on this subject.

With such facts, as we have stated above, before our eyes—of children from seven to thirteen years of age being kept on their legs for thirteen hours out of the four and twenty, while all the time they are ill-fed, ill-clothed, and miserably housed—surely it is high time that some change be soon effected. It may be true that in all manufactures there are times of distress, and that often the masters cannot afford to give high wages or allow short hours to those in their employ. It may be also quite true that, in the present day of restless competition, not only between the merchants of one country but between different countries, all striving for the same end, the only chance of success depends upon low prices, and these are only to be had by low wages on the part of the workmen, and small profits on the part of the masters.

Still these considerations, although worthy of attention, ought not to deter our legislature from interfering on so national an object, as that of the health of hundreds of its young inhabitants. Humanity, nay the dictates of ordinary policy, should prompt to the adoption of some means without delay. It cannot be a prudent, it cannot be a safe, state of things for a country to have a large portion of it population feeble, sickly, and decayed.—*Annales d'Hygiene Publique.*

This short statement will be read with interest. It shews that the state of the manufacturing districts in France is quite as bad as in our own country, and that the factory system is urged to as extreme a length. Children, from seven to thirteen years of age, kept at work for twelve and fourteen hours of the day, forced to walk some miles before and after work, ill-fed and often in rags—such a picture of misery! Oh! what will not cupidity make man do to his fellow-creatures. It is truly melancholy to think of all these things; and withal to hear, eternally dunned into our ears, eulogies of the amazing progress of civilization, refinement, charity and so forth, in the present day. We have shewn in another article in this Periscope, that insanity has increased with increasing knowledge and prosperity—alas! vice and misery too seem to follow in their train.

---

### Suggestions for the Improvement of the Morals and Manners of the Inhabitants of large Towns in Germany!!

Our readers will be probably surprised at reading the above notice; and they may well puzzle themselves with trying to discover what business a medical journal has to do with the doctrines of ethical science. Professor Wildberg, however, tells them that morality is but one department of medical jurisprudence (!)—the scope and object of which are not to be confined to the examination of those questions alone which relate to the health of communities, or to the detection of crimes, but should embrace a much wider field, and should afford a code of regulations for the general conduct and demeanour of the people!

We are tempted by another consideration to extract a few of the Professor's instructions—in order that our readers may judge for themselves of what sort of *stuff* a large portion of the periodical medical literature on the Continent is composed. The paper from which we borrow these notes is headed, "Remarks on some objects of medical policy, which, notwithstanding their importance for the health and lives of the inhabitants, are utterly neglected in many places."

1. In many districts, we are informed, there is a pernicious custom of crowds assembling before the house of the bride and bridegroom on the eve of the nuptials, and causing great noise and disturbance in the street, by screaming, knocking at the doors, and by all sorts of boisterous revelry. This evil ought certainly to be put a stop to (!) It might be very easily suppressed; and we are therefore surprised that the public authorities have so long permitted its continuance. (!!)

2. In most towns we find that adults as well as children are in the habit of sitting down and obeying the calls of Nature (hinsetzen und ihre nothdurft verrichten) in the streets and open places; and also that all sorts of rubbish, dead cats and dogs, &c. are often thrown down on the public highways. (We humbly suggest to our German brethren the example of their Parisian neighbours, who have the merit of having introduced, into every part of their matchless metropolis, the great public convenience of "cabinets d'aisance inodores (!)

3. The practice too of children being permitted to be running about the streets, and playing all sorts of games is highly reprehensible! These little urchins may unknowingly be the cause of mischief to themselves and also to passengers. This evil also might be very easily got rid of by a simple prohibition. (Auch diesem uebel wurde durch ein einfaches verbot leicht zu steuern seyn.)

4. The bathing of the youth in rivers and in the sea ought to be subjected to certain regulations, in order that the distressing accidents, which are occurring so frequently, may be prevented. With respect too to skating and to sliding on the ice, the public authorities ought to issue certain orders, cautioning the citizens against venturing on the ice, except it be sufficiently strong and able to bear them.

5. All idle and unoccupied men ought to be viewed and treated as useless and hurtful members (nutzlose und schadliche glieder) of society. A much sharper eye ought to be kept on this sort of people, than is usually done. They are not only *drones*, but positive *pests* of large towns.

6. The same remark is applicable to all the unmarried women, who are not engaged in some regular honest employment, but live by prostitution, (mit ihrem leibe gewerbe treiben.) The magistracy might do much to improve these women; and if all such attempts fail, they ought then certainly to be banished from the city. (!)

7. In all large towns there are numerous public houses, where beer, brandy, and other drinks are prepared and sold. Now all such places ought to be regularly visited and inspected by competent persons, whose duty it should be to examine all liquors exposed for sale, as well as to find out of what materials they are composed, and how they are manufactured. Similar regulations should extend to all butchers and dealers in meat. All the cattle should be examined, before they are slaughtered. We are favoured by our learned author with the following code of regulations. Every beast should be as thoroughly drained of its blood as possible; the *blowing up* of meat should be prohibited under a severe penalty; the hide, or, as it is called, the sward of pigs should be carefully cleared of all the bristles (!); and lastly, no meat should be cut up, or salted, until it has become thoroughly cool.

[Our readers have probably had enough of this puerile niaiserie; yet of such materials is a large portion of German journalism composed. Those who may wish to consult the original will find it in—*Wildberg Jahrbuch der gesammten Staatsarzneikunde.*]

---

Rheumatism, Nature or Proximate Cause of—shewn to be a Disease of the Fibrous, and not of the Muscular Tissue—Employment of Opium in its Treatment, &c.

The question, which we propose at present to endeavour to solve, is, what is the genuine seat and nature of Rheumatism. Is it really an inflammation of the *muscular* substance? or is it an inflammation of the *fibrous* tissue of the limbs, and joints, viz. the aponeuroses, fasciæ, articular, capsular ligaments, &c.?

The former of these two doctrines was almost universally adopted as true—indeed, without almost any dispute—by our forefathers. Rheumatism in nosological arrangements was generally designated and described as a Phlegmasia of muscle, just as Arachnitis has been used to denote an inflammation of the arachnoid membrane, or Peritonitis of the peritoneum.

The fallacy of this doctrine has of late years been very clearly established; and it is now very generally admitted by the best pathologists, *that the fibrous tissues are the genuine seat of rheumatic inflammation*—at least in the acute form of the disease.

We cannot indeed appeal, with as much confidence as we can in reference to other inflammatory disorders, to the results of morbid anatomy in proof of this position; as every one knows that uncomplicated Rheumatism very rarely proves fatal, and that, therefore, the physician can seldom examine the morbid changes which the disease may occasion.* It is however, to be noticed, that in the few cases, which have proved fatal, and in which there was a careful examination of the affected parts by dissection, the muscular tissue has been found to be, almost, if not altogether, quite exempt from any change or lesion; and that the most obvious and well-marked signs of morbid action—and even these, have in most instances been far from being satisfactory and "bien prononcés"—have consisted in thickening of the aponeurotic and capsular membranes, and in some slight changes, either as to quantity or quality, of the synovial fluid.

---

* We cannot however but think that hospital physicians in particular have neglected many opportunities, which they have, of carefully inspecting the appearances of the joints and limbs in patients, who may have suffered at some period of their lives from acute or from chronic Rheumatism. We know for example, that a large majority of the cases of Pericarditis occur in rheumatic patients; and yet in the pathological history of that disease, there is seldom or never any reference to the other morbid appearances, save those which are found in the chest.

M. Brachet informs us that Lyons and its immediate neighbourhood—where he practices—is noted for the great prevalence of rheumatic complaints.

The climate is very moist, and the soil is clayey and damp.

Being the physician to one of the large prisons there, M. B. has very ample opportunities of treating a vast number of cases; and he tells us that, after long examination of the subject, he is now convinced that Rheumatism is not a *muscular* but a *fibrous* inflammation.

Reasoning had, in the first instance, led him to this view of the subject. He could not satisfactorily explain to his mind how rheumatism could be so metastatic or transitory from one part to another, if the muscles—organs which are distinct and independent of each other—were the seat of the disease.

The fibrous tissue, on the other hand, being continuous, and, as it were, expanded over the whole body, we are at once prepared to expect that a morbid action in one part may be quickly transferred to another. This is a feature, which is strikingly characteristic of every form of Rheumatism; and certainly we cannot find any rational interpretation of it, if we regard it as a disease of muscular substance.

The mere circumstance of the pain being often seated in the muscular parts of a limb cannot have much weight on the mind of any pathologist, when he calls to mind that, wherever there is a muscle, there is either an aponeurosis, or fascia, or tendon, or periosteum—all of which *tissues* are strictly *fibrous*.

M. Brachet admits that, in a very few cases of rheumatism, the disease has terminated in formation of an abscess in the muscles. (When this rare occurrence does happen, it is usually in the muscles of the thigh.) He has seen two or three examples of this nature in his own practice; but, in every one of them, the purulent collection had taken place either in the subcutaneous, or in the intermuscular, cellular texture; and never in the muscular tissue itself. In one or two instances he has found pus around the periosteum.

We have not alluded to the striking sympathy of morbid action between the tissues usually affected with Rheumatism and certain serous membranes, more especially the pericardium, as affording another argument in favour of the *fibrous pathology* of the disease. That there is a certain affinity in structure, functions, and morbid liabilities, between the serous and fibrous tissues was ably illustrated by Bichat, in his great work on the membranes, and cannot be disputed by any one.

The French, indeed, with their accustomed vivacity of invention, have often pushed the *doctrine of analogies* too far; and we must confess that we are not yet disposed to regard the pericardium as altogether identical with a capsular ligament of a joint.

Is their structure quite the same? certainly not. Is their secretion quite the same? No. Are their pathological or morbid lesions quite the same? We are forced to answer again, No. A capsular ligament is rarely found to be invested with genuine coagulable lymph, such as we so often find on the surface of the pericardium after death. Whether the peculiar oily nature of the synovia, as has been suspected by some, prevents in some degree the persistance of this effusion, we cannot tell; although we must confess that we are never much inclined to explain vital phenomena, either in health or in disease, on merely mechanical principles.

But waving these considerations, no one can dispute the striking connexion between Rheumatism and pericarditic inflammation; and we think that M. Brachet is quite justified in alluding to this connexion, as one argument which suggests the real nature and character of the former malady.

"It is however of importance to remember," says M. Brachet, "that although rheumatism is in our opinion strictly and truly an inflammation of fibrous structure, it is by no means the only *form of* inflammation to which that structure is subject. Rheumatic inflammation is unquestionably different in many respects from ordinary, or, what is sometimes called, simple inflammation of fibrous tissue."

This admission on the part of M. Brachet is highly creditable to his practical judgment, and shews us that the generalising and exclusive doctrine of the Broussaian school — which this Journal has so uniformly opposed—do not now infect the French writers, as it did some few years ago.

In reference to rheumatic inflammation being of a peculiar or specific character, we surely do not require any other argument than that of the diseased action never terminating in one of the most frequent consequences of simple phlogosis, we mean suppuration.

M. Brachet does not pursue this subject any further, as the main scope of his paper is to point out those forms or varieties of Rheumatism, in which the use of opium, internally and externally, is of most service.

When the pain is limited to one or two parts of no great extent, frictions with a strong opiate liniment,* or the application of an opiate plaster will often suffice to dissipate the evil.

But when the pain is widely diffused, and affects perhaps both upper and lower extremities, the neck, &c., and when there is active synochal fever present, the first and most important step in such a case is to order a large general bleeding. As long as the fever continues, at least with any severity, we should abstain from the internal use of opium, as there may be considerable danger of inducing a metastasis of the diseased action from the extremities to the brain. It is when the pulse becomes soft and less full, and when the skin is disposed to be moist, that the administration of opium will be found to be of most decided efficacy. M. Brachet seems to prefer the morphia to any other preparation for internal use.

As an example of the dangerous effects of opium injudiciously administered in acute rheumatism, M. Brachet relates the following fatal case.

A man was seized, in the Spring of 1821, with a violent attack of acute rheumatism. All his limbs were affected with excruciating pain, which was greatly aggravated on the slightest motion. The pulse was rapid and full, and all the other symptoms of active synocha were present. This state had continued for three days, when M. Brachet visited him. M. B. urged the necessity of immediate venæsection; but, the patient refusing, he prescribed some mild diluents.

On the following day he again urged the importance of general bleeding, as the pains were as severe as ever, and the synochal symptoms were violent. Still the patient would not consent; and M. Brachet contented himself with using merely palliative means.*

Next day another physician was called in: he prescribed the free use of

* The formula for the liniment which we usually order is the following.

℞. Liniment. saponis comp.

Liniment. camphor. comp.

Tincturæ opii, āā ℥ss. Misce.

* We cannot indeed approve of M. Brachet's practice on the present occasion.

In the treatment of severe inflammatory disease, should it so happen that a patient or his friends decidedly object to the use of the lancet, it is not the part of a wise physician to be inactive in consequence.

There are other remedies almost as potent as even bleeding itself, in reducing high vascular excitement. Of these the preparations of antimony and of mercury are by far the most efficacious.

By repeated doses of emetic tartar, either alone or in combination with small doses of opium to prevent excessive vomiting, or with colchicum, we may very generally succeed in abating the most vigorous synocha; and by using some mercurial preparation freely, we act on the state of the circulating mass of blood, so as to *attenuate* the peculiarly fibrinous character, which is almost always present in acute rheumatism. Our own opinion decidedly is that mercury is, perhaps of all remedies, the most powerfully *antirheumatic.*—*Rev.*

opium. Coma supervened, and the patient died in twenty-four hours afterwards.

The caution is well expressed in the following paragraph from Stoll's writings: "Cave ab opio, maximé vespere propinquando; stante inflammatorio stadio non convenit ob aliena et anomala nervorum mala; at, fracto subinde, cauté quidem dari potest."

In some cases of very obstinate rheumatic pain, which has resisted the usual modes of applying opium, viz. liniments and plasters, M. Brachet has frequently succeeded by having recourse to the Endermic method. He usually employs one grain of acetate of morphia, sprinkled on the denuded surface of the affected part.

M. Brachet very justly remarks in reference to chronic Rheumatism that most erroneous ideas have hitherto very generally, and still do prevail on its real or proximate character; and consequently that the practice, which is pursued for its relief, is most vague and empiric. It has been far to commonly taken for granted that chronic Rheumatism is only a less acute form of the disease which we know by the name of acute Rheumatism; and that the morbid action in the two disorders is of the same essential nature, differing only in its severity and degree.

Now as acute rheumatism is always most obviously a phlegmasia, it has been concluded that chronic rheumatism must be so too; with this difference only, that the inflammatory action is of a much less severe and active character, and not sufficient to produce any febrile excitement of the system. The error—and be it remembered that it is one of great practical consequence—of this doctrine consists in its being only *partially* correct. That many cases of chronic rheumatism consist in a subacute degree of inflammation of the affected parts cannot be disputed by any one. Its symptoms and causes, as well as the nature of the means which are most successful in relieving it, place the truth of this beyond all doubt.

But it is equally true that numerous other examples of chronic rheumatism exhibit very different characters—characters which are utterly incompatible with the idea that it is a disease of inflammatory nature. For example, how are we to reconcile with this view of the question the occurrence of *so-called* rheumatic pains in many invalids, whenever their stomach or bowels are slightly deranged? or how are we to explain the very evanescent and temporary duration—perhaps only for an hour or two at a time—of these pains? And lastly, how is it that an inflammatory action should be relieved not only by internal stimulants, but by active friction of the affected parts?

These are questions which, we think, are almost quite incompatible with the doctrine that chronic rheumatism is *always* an inflammatory disease.

If this reasoning be correct, it must follow that various affections, dissimilar in character, symptoms, and curability, are inclosed under the general term of chronic Rheumatism.

It is most important that the young, and many too among the older, practitioners pay attention to the distinction now alluded to, in order that they may direct their treatment on more stable and successful principles, than they have hitherto done.

For example, chronic Rheumatism, when it is dependent upon a low or chronic degree of inflammatory action in the affected parts,—and this may be inferred when the pain is made worse by heat, friction, and exercise, when the parts are tumified and tender, when the symptoms are permanent and not subject to alternations of aggravation and abatement, when they have supervened on an attack of the acute form, and when they are more or less independent of the state of the bowels—requires for its treatment the use of leeches and soothing fomentations, or of the warm bath, the administration of some mercurial with or without opium and ipecacuan, of purgatives and diuretics, a low diet, and in short a moderate and regulated antiphlogistic regimen.

The other form of chronic rheumatism is decidedly rather of a *neuralgic* than of an *inflammatory* nature. It is almost unnecessary for us to do more than simply to call the attention of our readers to the subject, after what we have already said. It is in this form of the disease that the use of the stomachics and tonics,—always provided the bowels and kidneys have previously been brought into a healthy state—change of air, travelling, chalybeate baths and waters, &c., are so pre-eminently useful.

Along with these means, the greatest attention must be paid to clothing, not only indeed in this, but also in all the forms of rheumatism. The use of flannel clothing next the skin, Summer and Winter, is indispensably necessary.—*De l'emploi de l'opium dans les Phlegmasies, &c. Par M. J. L. Brachet.*

*Remarks.*—We have interposed a good many observations of our own among the preceding extracts from M. Brachet's memoir.

On the whole we approve generally of the sentiments which he has expressed; and we have been much pleased to find that so able a physiological writer is at the same time a talented practitioner.

He, like many other of the best physicians in France, has not allowed his mind to be misled by the exclusive doctrines of Broussaism:—the very admission that the inflammation of Rheumatism is not altogether analogous to a simple phlegmasia, and that there is a special *something* in it which gives the disease its peculiar character, is quite heterodox in the eyes of the so-called physiological physicians.

We ourselves have repeatedly exposed the fallacies of the Broussaist creed, not only in reference to rheumatism, but also in its application to fever and several of the phlegmasiæ.

M. Bouillaud, being one of its most zealous and able advocates, has exposed himself more than others to our remarks. His recent works on Medical Philosophy, on Rheumatism, on diseases of the Heart, and on medical Clinique have, all, been amply noticed in the pages of this Review, and have thus enabled us to expose the errors, to which any exclusive system of medical doctrine must necessarily lead its proselytes.

In taking leave of M. Brachet's Memoir on Rheumatism, we ought to observe that no mention is made of mercury, or of colchicum in the treatment of the disease. We regard both as very powerful agents in the cure of all rheumatic affections, chronic as well as acute. Many of the most stubborn cases of the chronic form will be found to yield to a course of the blue, or of Plummer's pill, taken at bed-time, and of an aperient mixture, such as the following, taken next morning.

℞ Infusi sennæ, ℥iij.

—— gentianæ comp. ℥ij.

Magnes. sulphatis, ʒvj.

Tinct. jalapæ, ʒiij.

—— cinnamoni comp. ʒiij.

Vino semin. colchici, ʒiss.

Misce.

Warm clothing, and the occasional use of a warm bath should be adopted at the same time.—*Rev.*

---

Cases Illustrative of Albuminuria, or Bright's Disease of the Kidneys.

The following cases are adduced by M. Forget, one of the professors at Strasbourg, as examples of structural disease of the kidneys, which, he believes, is invariably indicated by an albuminous state of the urinary secretion.

It may be necessary to caution our readers against an unreserved admission of this doctrine—that, whenever the urine is found to be albuminous by the action of heat, or by the addition of nitric acid or of corrosive sublimate, there is reason to suspect a morbid affection of the kidneys;—as it is admitted by most of the best physicians in this country that *albuminuria* (to use M. Forget's term) is not unfrequently present with diseases of other organs, and when there is no good reason to suppose that the kidneys are affected. Drs. Wells and Blackall, many years ago, pointed out the frequent existence of this state of the urine in acute dropsy, more especially in that form of the disease which often supervenes upon scarlatina and other exanthematous diseases; and more recently Dr. Darwall and some other writers have assured us that they have repeatedly detected the same chemical condition in dropsies connected with diseases of the heart.

Although therefore *albuminuria* cannot be considered—as M. Forget seems to suppose—as invariably characteristic and indicative of disease of the kidneys, we may mention that, whenever this state of the urine is present, unaccompanied with dropsical symptoms, and more especially in a drooping or cachectic constitution, there is reason to suspect the kidneys as the seat of some morbid process. Not a few of those obscure chronic affections, which prove fatal without exhibiting, during any period of their course, any very marked or pathognomonic symptoms, have been found on dissection to be connected with, if not immediately dependent upon, a diseased state of the kidneys.

Dr. Johnson, the senior editor of this Journal, has recorded several interesting cases of this description. With these preliminary remarks, we now proceed to narrate briefly some of the most interesting observations from M. Forget's memoir.

CASE 1.—*Anasarca; albuminous urine; granular affection of the kidneys.*

An unhealthy semi-idiotic woman, 30 years of age, was admitted into the Strasbourg Hospital, in consequence of general anasarca.

There was an obscure fluctuation in the abdomen; but there was no reason to suspect disease of the liver, spleen, or any of the thoracic organs. The urine was scanty, but of a normal appearance.

By applying heat, or the nitric acid to it, a copious flaky white precipitate was formed.' Medicine made no impression upon the symptoms. She was tapped, and from eight to nine *litres* of serum evacuated from the abdomen. Diarrhœa and bronchitis came on; and the patient died in six weeks after her admission.

*Dissection.*—Thoracic viscera healthy. The peritoneum was vascular, sprinkled over with pseudo-membranes, and 'criblée' with numerous white tubercular granulations. The omentum was agglomerated together into one mass. The mucous membrane of the intestines was every here and there highly injected. The liver and spleen were healthy. The left kidney was slightly enlarged; its surface was whitish or of a marbled red or grey colour, and uneven; the cortical substance was pale, bloodless, and granulated with small white irregular spots. The right kidney was double the size of the other; its surface was greyish and sprinkled over with sanguineous patches; its texture was soft, and very granular; the cortical substance was yellowish and somewhat degenerated.

CASE 2.—*Anasarca; albuminous urine; recovery.*

A youth, after a week's indisposition, became affected with œdema of the limbs. There were no symptoms indicative of thoracic or of abdominal disease. He was bled to a small amount, and then treated with diuretics. A few days afterwards the pulse was found to be somewhat stronger and more rapid, and the heart beat with more than usual force. He was cupped over the sternum. At this period, the urine was examined, and ascertained to be albuminous, when nitric acid was added. M. Forget pronounced the case forthwith to be one of 'granular affection of the kidneys.' The antiphlogistic treatment was rigorously persisted in; and in the course of a month the patient was pronounced to be quite well; the anasarca having disappeared, and the urine having lost its albuminous character.

*Remarks.*—It is altogether conjecture on the part of M. Forget to call the preceding case of disease of the kidneys. The patient never, even on minute enquiry, made any complaint of uneasiness in the loins, &c.; and the mere circumstance of the urine having been albuminous is certainly not, in our opinion, sufficient to warrant such a diagnosis. Dr. Darwall no doubt would have adduced this case as confirmatory of his doctrine that *albuminuria* is frequently co-existent with dropsy, connected with affections of the heart.—*Rev.*

CASE 3.—*Anasarca; albuminous urine; cure of the dropsy; persistence of the albuminuria.*

A poor sickly youth, 17 years of age, after exposure to cold, was seized with general lassitude, cough, dyspnœa, and œdema of the legs. The urine was rather scanty, but not high-coloured. He was bled to eight ozs. and treated with digitalis and squills. Four days afterwards the urine was examined and found to be very albuminous. He made no complaint however of any uneasiness in the loins, even when firm pressure was made. He was treated with cupping of the loins, the use of vapour baths, frictions with the tincture of squills and digitalis, &c. In three weeks the dropsical symptoms had ceased; but he was still tortured with cough and other bronchitic annoyance. The urine too, even when he left the hospital appeared to be pretty well, continued to be very decidedly albuminous, when treated with nitric acid. M. Forget predicted that the dropsy would return, but he heard no more of him.

*Remarks.*—This case has certainly more right to be deemed one of *suspected* disease of the kidneys than the preceding. The persistence of the *albuminuria* is always an unpleasant symptom. —*Rev.*

CASE 4.—A woman, 43 years of age, was admitted the second time into the hospital with symptoms of dropsy of the limbs and abdomen. She was suffering also from thoracic distress which indicated an effusion of fluid into the cavity of the pleura; and the sounds of the heart, although not decidedly abnormal in character, were diffused over a greater extent than in health. The urine was scanty and very albuminous. She had slight uneasiness in the loins. She continued under treatment for nearly three months, but nothing seemed to have any decided benefit.

On dissection, the cavities of the pleuræ were found to contain a large quantity of serosity. The heart was nearly normal. None of the abdominal viscera were decidedly morbid, with the exception of the kidneys. Both were somewhat enlarged, and uneven and mottled with seemingly ecchymosed spots on the surface. When stripped of their *tunica propria*, which adhered rather loosely, the surface exhibited numerous small whitish granulations; the cortical substance was pale, yellowish and granular, and the tubular portion was of a deep livid colour.

The fifth and sixth cases are not very satisfactory. In the former the patient complained of pneumonia, without any symptom of dropsy, and the kidneys were found to be slightly granular. In the latter also the patient was labouring under pneumonia; but this was complicated with anasarca; the urine was very decidedly albuminous; and yet the man recovered, at least for the time. M. Forget anticipated a speedy relapse, in consequence of the *albuminuria* continuing.

The seventh case is especially interesting, as it affords an indisputable example of most complete *albuminuria* during life, when the kidneys were found to be 'parfaitment sains, sans anemie ni granulations.' The patient, a middle-aged woman, died from disease of the heart—(the heart was generally hypertrophied, and the mitral valve particularly was ossified and contracted)—which had produced anasarca and ascites.

M. Forget acknowledges that the result of this case has very much surprised him, as he had been led to suppose that, '*whenever there is dropsy with* albuminous urine, the kidneys are always diseased.' Had he been better acquainted with English medical literature, our author could not have fallen into this mistake; but like most of his countrymen, M. Forget is apt to be far too exclusive and one-sided in medical doctrine.

The eighth case too comes in support of the views we have inculcated—viz. that albuminous urine is not invariably the sign of disease in the kidneys. In it the patient died of heart-disease; and the kidneys 'n'etaient pas sensiblement alterés.'

In the 9th case, the patient had been long suffering from all the symptoms of heart-disease. She became dropsical and died. The urine had been for a length of time very albuminous. On dissection the heart was found to be much hypertrophied and its valves partially ossified. The kidneys were decidedly granular.

These cases therefore very pointedly illustrate the frequent co-existence of *albuminuria*, with diseases of the central organ of circulation.—(*Gazette Medicale de Paris*), *Sept.* 1837.

---

### PATHOLOGY OF PUERPERAL FEVER (METRO-PERITONITIS). By Dr. NONAT.

Dr. Nonat is one of the physicians of the Central Bureau of Hospitals, and had an opportunity of witnessing many cases of this distressing malady in 1831. The memoir, which he has written on the subject, was deemed of sufficient importance to be printed by the Society of Medicine.

We shall give a short summary of the anatomical appearances, which he discovered on dissection.

1. *Lesions of the Uterus.* The outer surface of this organ was usually injected; sometimes over its whole extent, at other times in patches; more distinctly at the lower part than towards the fundus, and at the sides more than in the middle. Occasionally ecchymosed spots were observed around the os *tincæ;* but this appearance, be it remembered, is not unfrequently found, when no puerperal fever has ever existed.

On the inner surface of the uterus, the debris of the lochial secretion and of the placenta was found. This was of a semi-fluid pseudo-membranous appearance, of a yellowish brown colour, and often most fetid and offensive. On removing this *detritus,* the surface of the uterus itself was usually found to be softened or even pulpy, and of a yellowish, or of a livid hue. In some cases, the entire substance of the organ was soft throughout, and readily tore on stretching it; while, in other cases, the *ramollissement* affected chiefly the cervical portion.

Besides these morbid appearances, a purulent infiltration occasionally pervaded the parietes of the uterus, more especially round its cervix. In not a few instances, small abscesses (foyers) were found imbedded in the uterine substance;—these were most numerous towards the lateral parts, near to the attachment of the ligaments, in the cellular texture surrounding the cervix uteri. Two cases of genuine gangrene of the uterus occurred in my researches.

2. *Lesions of the Uterine Lymphatics.*—It is well known that these vessels very greatly enlarge during gestation. When filled with any coloured fluid, it is very easy to trace them; as some attain the size of writing-quills. On leaving the uterus, they collect at the line of attachment of the broad ligaments, and form four or five main trunks, which accompany the spermatic vessels, and proceed on to the lumbar and sacral glands.

In several cases these vessels and glands were filled with a white, inodorous fluid, similar in every respect to the pus of a healthy abscess.

They could be readily traced, along their whole extent, and exhibited numerous alternate dilatations and contractions. The lymphatic vessels, distributed in the substance of the uterus itself, never presented any appearance of purulent matter. The lumbar glands were often enlarged, infiltrated with pus, soft and easily lacerable. The *vasa efferentia* from these glands were not unfrequently injected with pus; and thus their numerous plexuses around the large blood-vessels and along the bodies of the vertebræ were distinctly and beautifully visible. In one case only did the Thoracic duct, to the extent of two or three inches of its course, contain a purulent fluid: in all the others, its contents were quite transparent.

M. Nonat is of opinion that the purulent contents of the lymphatics are, in many cases at least, generated in these vessels themselves, in consequence of an inflammatory action of their parietes, and not always absorbed from the substance of the uterus or from the cellular tissue, as Velpeau, Duges and Duplay imagine.

This point however in the pathology of puerperal fever is still undecided; and perhaps it is a matter more of curiosity than of much practical importance.

It is not improbable that the mere presence of a foreign fluid, such as pus, in the vessels excites an irritative action in their parietes; and the effect of such irritation may very possibly be to increase the amount of their purulent contents.

3. *Lesions of the Uterine Veins.*—These vessels, in numerous cases, presented all the appearance of inflammation. They were larger than in health; their parietes were very vascular, of a brownish red colour, thickened and more or less softened. In some cases, the veins on one side only of the uterus were diseased; but more frequently the inflammatory lesions had affected the vessels on both sides, and along their whole extent from the uterus to their *embouchure* in the vena cava, or one of the renal veins.

In a few cases, the morbid action was traceable in the hypogastric, iliac, and crural veins; and occasionally the vena cava itself was not exempt. The contents of the inflamed veins were usually of a brownish or yellowish colour, varying in consistence from that of ordinary pus to that of sanies. In the smaller branches little or no blood was discoverable; but in the larger ones the pus was mixed with sanguineous clots; and, in proportion to their distance from the uterus, the quantity of blood to that of pus increased. In one case only did the contents of the veins emit an offensive gangrenous odour, similar to that of the substances found in the cavity of the uterus.

The diseased veins were generally much thickened in texture, and their internal surface was injected and occasionally lined with membraniform deposits: their coats were in most instances sensibly softer than in health.

M. Nonat tells us that he traced the diseased veins very generally to that point of the uterus, where the placenta had been attached; and in all his examinations their orifices were plugged up with a coagulum. In not one case, were they found open or gaping on the internal surface of the uterus. He also mentions that such was the state of the veins, at some little distance from the seat of the malady,—so plugged up with coagula of blood—that it was quite evident that the purulent contents of the diseased branches never could be conveyed into the circulating mass; and hence he very fairly concludes that the typhoid symptoms of puerperal fever cannot be attributed to the corruption of the blood, in consequence of the absorption of pus into its current, but are altogether referrible to the effects of the local mischief on the general powers of life.

M. Nonat is of opinion that the pus in the veins is generated in these vessels, and is not the mere result of absorption from the cavity or substance of the uterus, as some writers have alleged.

4. *Lesions of the Peritoneum.*—In a large majority of fatal cases of puerperal fever, this membrane is found on dissection to exhibit more or less distinct marks of inflammatory action.

Besides well-marked vascularity, either continuously for a considerable extent or in patches, we frequently observe depositions of pseudo-membrane on its surface, and also an effusion, variable in quantity from eight or nine ounces to several pints, of a sero-purulent fluid in the cavity of the abdomen. This fluid often contains numerous flocculi, not unlike to the white of egg.

Other serous membranes besides the peritoneum are often found similarly diseased in many cases of metro-peritonitis.

Of these the pleuræ are most frequently affected: they are inflamed, partially coated with membraniform exudations, and their cavities contain a sero-purulent effusion. The synovial membranes of the large joints also are, in not a few instances, equally morbid.

M. Nonat has in no case discovered any well-marked traces of disease in the membranes of the brain, or in the pericardium.

And here it is especially worthy of notice that the above-mentioned lesions may be found most extensively in the serous membranes of the abdomen and of the thorax, and also in the synovial capsules, while no traces of inflammation, either of the veins or of the lymphatics of the uterus are discoverable. This fact, therefore, affords an incontrovertible objection to the idea that the disease of these vessels is primarily and essentially the source of mischief in puerperal fever.

5. *Lesions of the Digestive Tube.*—The researches of M. Nonat most satisfactorily establish the fact that, in the majority of cases of puerperal fever, there are no important lesions of the intestinal canal.* The mucous membrane may be slightly vascular in points or points; but it is most generally pale; and its glands, aggregate as well as solitary, are healthy. The only morbid appearance, says our author, which seemed to me to be constant, was the presence of a large quantity of yellowish or green bilious fluid, which filled the stomach and intestines.

We ought however to add, that in cases of puerperal fever which have lasted very long, we do sometimes meet with certain lesions of the intestinal tube—such as redness, softening, and partial effusion of lymph—which are indubitably of an inflammatory origin.

Such are the most frequent and important morbid phenomena found in fatal cases of Metro-peritonitis. There are others of occasional occurrence, which deserve to be enumerated. Thus, for example, M. Nonat found in one case a large quantity of pus in the tendinous sheaths of the forearm; in another, the mediastinum and portions of the lungs were infiltrated with pus; and in a third case, in which the iliac veins were remarkably inflamed, there were several vomicæ dispersed through the pulmonary tissue.

M. Nonat remarks that, as well as he can judge from his researches, *secondary abscesses* are on the whole of less frequent occurrence after uterine phlebitis, than after the other forms of venous inflammation. Whether this difference is attributable to the circumstance of the visceral veins being more readily and completely obstructed by coagula—so that the introduction of their morbid contents into the circulating current is less easy and frequent—than the veins of the extremities, we are not warranted in deciding.

Lastly, M. Nonat alludes to the state of the blood itself, as one of the pathological characters of puerperal fever. He confesses that hitherto he is not prepared with any satisfactory observations on this interesting subject; and suggests the probable advantage of microscopical researches to aid our inquiries. The blood in this disease is almost always thin, and in, what is called, a dissolved state. It does not readily coagulate; the clot being usually very soft and imperfect, and the same being of a sanguineous colour.—*Revue Med.*

The memoir of M. Nonat is a very valuable one. The obstetrical reader will do well to take a memorandum of where it is to be found. It is contained in the Sept. No. of the Revue. It deserves to be printed as a monograph.—*Rev.*

* Might not this circumstance open the eyes of those exclusive pathologists, who can see nothing in typhoid fever, but a mere inflammation of the intestinal canal? Surely the analogy between puerperal and typhoid fevers is sufficiently close to warrant the physician in expecting *some* degree of resemblance in their pathological phenomena.

### On Phlebitis after Surgical Operations.

It is a curious fact in the history of this form of Phlebitis, that we sometimes find veins at a distance from the wound inflamed and filled with pus, while the intermediate vessels are exempt from these morbid charges. The following is an example.

A man received a gun-shot wound in the knee-joint during the disturbances in July 1830: the limb was amputated. A fortnight afterwards the patient exhibited symptoms which caused suspicion of *purulent infection:* he died in a few days.

The crural vein at the Fallopian ligament, and also the external iliac vein were thickened, coated with lymph, and contained purulent matter. The crural vein between this point and the wound, as well as its branches, were quite healthy; and its divided end was plugged up with a clot of blood. The periosteum of the os femoris, for a considerable extent, was loose and detached from the bone.

Dr. Blandin's researches throw some light upon such cases. He is of opinion that the origin of phlebitis, in such cases as we are now treating of, is very frequently in the veins of the osseous texture.

It is of great importance, therefore, that the attention of the surgeon be directed to this point in the post-mortem examination of such cases; more especially when the veins at a distance from the wound are found diseased, while the extremities of the divided vessels are exempt from any morbid change.

M. Blandin says, that it is only after amputation of the limbs, where bones have been sawn through, that the occurrence of *distant* phlebitis is discovered; and that, when the veins become inflamed after other surgical operations, in which the soft parts only are cut, the morbid action may always be traced from the divided extremities of the vessels.

Now, what is the cause of this difference? Perhaps it is to be sought for in the peculiarty of the veins of bones;—being not capable of closing or contracting upon their cavities as the veins of the softer parts.

This phenomenon is, M. Magendie assures us, readily confirmable in the living animal.

Every wound, involving the section of a bone, must continue to ooze out blood for some time,—if not from the soft, at least from the osseous surface. How distinctly is this the case in wounds of the cranial bones.

By attending to this peculiarity in the veins of osseous parts, we may explain how inflammation, when it attacks the surface of a wound, may very readily extend along these vessels. Besides; this patent state of the veins of bones must render them more ready to absorb any purulent or other discharge. We must not, however, push these conjectures too far. It is sufficient for our purpose to direct the attention of surgeons, in examining the pathology of phlebitis and secondary abscesses after amputations, &c. to the condition of the osseous veins. In some cases we find that the medullary substance of the divided bone has been converted into a *putrilage,* and its canal filled with an offensive pus. M. Blandin mentions that he met with these morbid appearances six different times, and M. Nonat alludes to one remarkable case.

This latter gentleman has come to the conclusion that pus is very seldom, if ever, absorbed by the veins into the system, so as to give rise to that train of symptoms usually designated by the term *purulent infection.*

He is of opinion that this most fatal state is rather owing to the formation of pus in the veins themselves, in consequence of a previous inflammation of their inner surface.

Some physiologists have attempted to prove the former doctrine by alleging the results of certain experiments, wherein purulent matter was injected directly into the blood. But granting that a serious disturbance of the health may be—as indeed all analogy might lead us to expect—induced in this manner, the question as to purulent absorption is still left undecided. We are therefore led to the conclusion that the train of formidable symptoms, which follow sometimes amputations of the limbs, at other times delivery and so forth, is attributable not to the direct absorption of purulent matter into the system, but to inflammation having attacked the inner surface of the veins, and having, as generally happens, terminated more or less extensively in suppuration.—*Revue Medicale.*

---

On a peculiar Affection of the Fore-arm—Inflammation of the Sheaths, &c. of certain Muscles. By M. Maingault.

There is an affection of the lower extremity of the fore-arm, which is scarcely alluded to in surgical works, and which nevertheless is sufficiently important to merit attention—we mean the inflammation of the *grooves* on the radius in which the *Radiales externi* muscles play.

Boyer, in his Treatise on Surgery, has devoted only a paragraph or two to the mention of this complaint, when treating of fractures of the lower extremity of the radius. His words are: "It is well to observe, in reference to this sign (crepitation) that persons, who use their hands much in laborious and fatiguing work, are subject to a peculiar affection of the cellular tissue surrounding the *extensor brevis,* and the abductor *longus pollicis,* in which these muscles become more prominent than usual, and give out on pressure a crepitating-like sound, or one like that which we hear when starch is pressed between the fingers. This sensation is indeed very different from that of genuine crepitation produced by the rubbing together of the broken end of a bone, and cannot well impose on the cautious surgeon."

M. Maingault has seen many cases of this affection, and seems to have examined it with peculiar care.

Boyer, he says, is quite right in stating that it is common in persons who work much with their hands; but, he adds, it is less from the actual fatigue, than from the nature of the work, that the affection seems to proceed.

Those persons are especially subject to it, who have to press hard upon the substances on which they have to work, and who moreover have to use a twisting movement of their wrist-joints, so that the hand and the forearm are kept in a forced pronation, and the extensor and abductor muscles of the thumb are at the same time powerfully contracted. These movements tend to stretch the fibrous sheaths which envelop these muscles, and also to irritate the synovial membranes of the grooves, on the bone, in which they play.

The workmen, most subject to this affection, are hat-makers, curriers, harness-makers, plasterers, dyers, and washing people. Youths also, who engage much in gymnastic exercises, sometimes suffer from it.

Persons who write a great deal, painters who use the brush for a length of time, and those who are obliged to keep their hands long in a constrained position, often experience a sense of weariness, amounting almost to pain, on the outer side of the right wrist-joint and fore-arm. Frequent repetition of these exercises will induce a swelling, and a certain degree of induration of the parts; and hence we not unfrequently observe in such persons a longitudinal firm prominence or bumb, extending from the metacarpal bone of the thumb to the lower third of the radial side of the fore-arm.

This prominence occasionally presents the appearance of two cords, separated by an intervening groove or depression. This depends upon the anatomical arrangement of the tendons; those of the short *flexor* and of the long *abductor* of the thumb lying in one groove of the bone, and that of the long *extensor* lying in another, somewhat behind the former.

The affected parts are sometimes exceedingly tender after exercise, so that all movements of the thumb are nearly impracticable. When the tenderness, &c. have subsided, we may then often perceive that peculiar crepitating-like sound on pressure, which Boyer has alluded to. In addition to fracture of the lower extremity of the radius, there is one form of luxation of the wrist-joint, which may possibly be confounded with the affection of the tendinous grooves and sheaths now described. M. Maingault mentions that when children are lifted up by one hand, for the purpose of carrying them over a puddle or stream, the radius may be slightly displaced from its play on the ulna, in consequence of the forced pronation which then takes place. Such an accident however may be distinguished from the affection, of which we have been treating, by several signs; as, for example, by the fixed position of the hand in a state of pronation, and by the projection of the end of the radius.

But to return to the proper subject of these remarks, we may mention that it may possibly be mistaken for a rheumatic or gouty inflammation of the wrist-joint. But the persons most subject to the former are not exposed to the ordinary exciting causes of gout at least; and moreover the strictly local seat of the swelling and pain would necessarily make the medical man pause in his diagnosis.

The parts which, according to M. Maingault, are chiefly affected, are the tendons of the muscles on the outer side of the radius, the fibrous grooves in which these play, the synovial membrane which lines them, and the cellular tissue which invests all.

With respect to the treatment of this affection, rest of the part and the use of a roller, passed somewhat firmly round the wrist, will generally suffice to remove it. In some troublesome cases, it will be well to apply a pasteboard splint on the outside of the joint, and retain it in its position by a few turns of a bandage.—*Revue Medicale.*

[We should consider that strapping the part with the emplast. hydrargyri spread on leather might be very useful, and least troublesome.]

### On the Utility of very large Blisters in White Swellings and other Tumors of the Joints.

M. Velpeau assures us that he has obtained the most gratifying success from blistering the entire knee-joint—and that too repeatedly—in several cases of white-swelling. The ordinary practice of applying a succession of small blisters is not nearly so efficacious, and on the whole is much more painful. He has used it, he says, in upwards of 200 cases; and almost always with indisputable benefit. (As a matter of course, we may reasonably suppose that, in the majority of these cases, the disease was not real white-swelling, but only a chronic inflammatory enlargement of the joint.)—*Rev.*

This *vesicatoire monstre* is to be kept on the part for 24 hours, and then dressed with any simple ointment spread upon blotting-paper.

An English servant was admitted into La Charité Hospital under the care of M. Velpeau. The right knee was enormously swollen; the distended capsule appeared to reach upwards nearly to the middle of the thigh, and downward to the spine of the tibia. The inequalities of surface, the fungoid aspect, and the indistinct deep-seated sense of fluctuation—all these signs indicated the existence of a most serious *arthropathy.** The disease had lasted for ten months, and had resisted various treatments at different times.

M. Velpeau ordered a blister, of a foot in length and ten inches in width, to be applied round the enlarged joint. The report closes by stating that he, M. Velpeau, saw the patient only three times more; and that a perfect cure was effected in less than two months.

Another case, more remarkable as having been pronounced by many excellent surgeons in Paris as incurable, save by amputation, is then described. It occurred in a woman, in whom the right knee was larger than the head of an adult, and presented "tous les caractères du *fongus articulaire.*"

The case had been adjudged, some time before, to one of the candidates for the vacant chair of clinical surgery; and it was then given as the opinion of most who examined it, that it was probably beyond all remedial treatment. And yet two *monster blisters* have sufficed to effect an entire cure of this immense swelling. The effused fluid was rapidly absorbed, and the joint recovered almost its normal dimensions.

---

* In this country, at least, such a case would certainly not be regarded as one of *white-swelling.* It seems to have been one of simple chronic inflammation, which had terminated in large effusion into the cavity of the joint.—*Rev.*

[Is it not strange that such a case as this could possibly have been deemed one of malignant disease by any experienced surgeon? and yet here we have a man of acknowledged repute, M. Velpeau, lending himself to such a statement. If true, it gives us a sorry opinion of the diagnostic talent of many Parisian surgeons.—*Rev.*]

So great had been the distention of the capsular ligament, that the joint could be nearly dislocated backwards at will, for some time after the fluid had been absorbed.

In addition to these large blisters, M. Velpeau talks praisingly of various pommades, &c. as powerful means to dissipate articular swellings; but as we have no intention at present to discuss the subject of *arthropathies*—(such is the new term for diseases of the joints) we shall pass them over.—*Archives Generales.*

---

### Cases of Fracture.

Case 1.—*Fracture of the Patella by Muscular Effort.*—A man, 49 years of age, was admitted into the infirmary of the Hôtel des Invalides, on the 22nd of June. On the preceding day, when coming down the steps of one of the *trottoirs* at the Pont Neuf, he missed his footing. Feeling that he was about to fall back, he made a violent effort to save himself by throwing the body forwards. He succeeded so far as to come down fairly on his buttocks, without his head touching the ground. But when he attempted to get up, he found this to be impossible. When brought to the hospital, the right patella was discovered to be fractured transversely. The man sustained no other injury.

This case confirms the remark made by Boyer, that, in the majority of cases of fractured patella, the fall is the *effect*, and not the *cause*, of the accident.

Case 2.—*Fracture of the Femur, Spontaneous.*—M. Olin, a sub-lieutenant, was admitted into the hospital on the 18th of June. He had been for a considerable length of time affected with paralysis of the left lower extremity.

On the day previous to his admission, M. Olin, while lying in bed, began to exercise his limbs in a variety of movements, with the view of giving pliancy and elasticity to the weak joints. He had been long in the practice of doing this every morning before he rose.

While attempting to bring the paralysed limb up towards his right shoulder, he suddenly heard a snapping, as if something had given way, but without feeling any pain. The limb however he found to be deformed, and when he made an effort to rest on it, he found that it gave way under him.

When brought to the infirmary, the surgeon discovered a fracture of the upper extremity of the femur, just below the trochanters.

There had been no reason to suspect at any time disease of the structure of the bone itself.

M. Perier, the surgeon of the ward, judiciously remarked that, in consequence of the paralysis of the limb, the energy of the nutritive process had probably been for a length of time in an impaired condition, and that the osseous tissue might thus have become more friable than in a state of health.

The fractured limb was placed in the immoveable apparatus of Baron Larrey; substituting however, as a varnish, a concentrated solution of starch in place of the alcoholic mixture recommended by this celebrated surgeon.

The former, when cool, becomes still more adhesive than the latter; and moreover it has the advantage of being easily removed, by merely wetting it with hot water.—*Gazette des Hôpitaux.*

---

### Poisoning by Nitric Acid:—Subsequent Induration of the Pylorus and Death.

A man 34 years of age, swallowed a wine-glassful of nitric acid, for the purpose of self-destruction. The greater portion of it was immediately rejected by vomiting. However an intense gastritis supervened, and was combated with active bleeding, the application of numerous leeches, the internal use of calcined magnesia, &c.

He was taken to the La Charité on the 8th day after the accident, and Professor Bouillaud continued the same course of treatment; and with such decided beneficial effects, that the patient was able to leave the hospital in three weeks. But within a month from his discharge he returned, in consequence of the severe pain he experienced along the tract of the gullet and at the epigastrium, and of frequent vomitings, especially after taking food. To these symptoms were added signs of an acute ascites; and although they were speedily subdued, the patient gradually sunk, and died about the middle of September—three months from the date of the poisoning.

On dissection, the pylorus was found so contracted, that its diameter did not exceed a line or two; and its contraction extended for an inch and a half along the commencement of the duodenum; the mucous membrane exhibited patches of redness, and of *ramollissement,* and also several cicatrices of ulcers; the subjacent tissues were scirrhous, and of a lardaceous aspect.

---

### REMARKS ON EPIDEMIC DISEASES.

M. Villeneuve published a statement some time ago, of his extended investigation of the epidemics which have been known in France from the year 1771 to 1830, and of which reports, amounting in number to 1160, had been sent to the Academy. The great arrear of business, which the Royal Society of Medicine, and the Society of the Faculty of Medicine at Paris, had bequeathed to their successor, the present Academy, had made such an enquiry, as that of which the report was now presented, quite necessary; seeing that it has always been the duty of these three respective institutions to supply the government with all necessary information on the important subject of Epidemic diseases.

In the prosecution of the enquiry, M. Villeneuve experienced considerable difficulty in determining the specific nature of the epidemics which have prevailed since the year 1771, and consequently in arranging them in their respective groupes.

This arose, partly from the imperfect descriptions which had been given of them, and partly from the varying and undefined nomenclature which had frequently been adopted.

By far the most universally alleged causes of epidemics, to judge from the numerous reports which were examined, appear to be

1. Alterations in the condition of the atmosphere — induced by exhalations from ponds, marshes, peat-lands, dung-hills, burial-grounds, &c.

2. Insalubrity of dwellings—whether this proceeds from a faulty construction of these; from the accumulation of wares and articles of provision; from the uncleanliness of the inhabitants; or from their being too much crowded together.

3. The bad quality of foods and drinks. And here it must be observed, (however painful the report will be) that it is but too true that, at various periods in certain districts of "notre Belle France" the most necessary articles of subsistence have been not unfrequently wanting, and the people have been forced to eat the very grass of the fields, and have not even had wholesome water to drink. (!)

4. Excess of labour, and unwholesomeness of some employments.

5. Debilitating moral influences, ignorance, and certain pernicious prejudices and customs.

In illustration of the last-named circumstance, our author mentions that in the department of the Oise, during the prevalence of the "Suette," or sweating sickness, which prevailed there in 1821, it was a very general practice to confine numerous patients together in close and heated chambers, to load them with bed-clothes, and ply them with stimulating drinks, &c., for the purpose of preventing the accession of sleep;—for this was deemed to be almost always fatal!

### Cachexia Aquosa in Egypt.

This disease is endemic among the poor and working classes in Egypt. It usually commences its ravages after the inundation of the Nile, attacking chiefly those, who work in the lands which are or have been overflowed. Its earliest symptom is an excessive weakness and languor of the whole system, and its general effects are somewhat akin to those which occur in scurvy, chlorosis, and elephantiasis. Very frequently it is complicated with dysentery; sometimes the local affection precedes the constitutional cachexy.

The only treatment which is of any avail, is the removal of the patients from the unhealthy localities, and the ample allowance of light nutritious food. Belladonna was occasionally useful, to allay the palpitations which distressed many of the sufferers.

The same description of disease prevails among the sheep, which feed in the low and marshy grounds of Egypt.

The inhabitants attribute it to their eating a deleterious plant, [the name is not given.] It bears some affinity to what is called the *rot* in Europe, but is never accompanied with the *staggers.* From 15 to 16,000 sheep are annually destroyed by this cachexia.

---

### Raw Potatoes in Scurvy.

A surgeon of a French South-sea whale ship informs us, that the health of the crew began to decline very much on the homeward-voyage. "Un matin," says the quick-eyed doctor, "je m'aperçus que le visage des hommes palissait: ils etaintenervés:" but Heaven was kind; for the symptoms of this "affection redoutable furent aussitot combatues que remarqués."

The remedy was novel: a bucketful of raw potatoes was ordered to be put on the deck at the foot of the mainmast, every morning *at eight bells,* and the sailors were advised to eat freely of them, as they would do "des meilleurs fruits d'espalier!" What was good for the men, was not bad for the masters. The captain and doctor assure us that they themselves found "à ces tubercules un saveur agreable, fraiche à la bouche et sucrée." The effect was, as we might have guessed, most marvellous. The bucket was filled every morning, and not one *pomme de terre* was ever to be seen by the evening. Monsieur continued to enjoy his daily feast till the ship arrived at Havre, after having been ten months at sea, without touching at any port. In conclusion, says our author, "on peut considerer le pomme de terre comme la Providence des navires dans les voyages de longs cours."—*Annales d'Hygiene, &c.*

---

### Intense Cephalalgia, induced by the Presence of Grubs in the Frontal Sinuses.

A woman presented herself recently at the hospital in Sienna, complaining of intense headache. The pain was most severe over the forehead; and often it was so distracting that she became delirious.

She said that, some time before, a common fly had got up one of her nostrils; but, whether it ever came out again, she did not know. The physician in attendance, suspecting that there might possibly be some of its ova deposited in the nasal cavities, advised her to fumigate her nostrils with the vapour of some anthelmintic substances. Judge of her surprise, when, a few hours afterwards, she found that several full-formed grubs were discharged. Upwards of fifty came away during the next week. These grubs were at once recognised to be those of the common flesh-fly, *(mosca di carne.)*

To prove that there was no mistake, several of them, being kept in favourable circumstances, passed from the state of chrysalis to that of a perfect fly The woman was at once relieved from all her sufferings.—*Bulletin Med. Belge.*

---

### DYSMENORRHŒA RELIEVED BY THE FUMES OF CARBONIC ACID.

Every physician is aware that some females suffer most severe pains in the uterine region, for one or more days before each appearance, and not unfrequently also during the continuance of the catamenial flow. Young girls residing in large towns are perhaps more subject to this distress than any other females;—their systems being often unusually irritable, and this excess of irritability being very generally associated with constitutional weakness. It is a common remark that such girls menstruate earlier in life than such as are robust, and those who reside in the country. Under these circumstances, marriage will often aggravate the dysmenorrhœa;—the generative organs being apt to be so highly excited by coition, that the accustomed monthly discharge, intended no doubt by Nature as a means of local relief, is either stopped altogether, or is only very sparing and uncertain. The treatment of such cases is often extremely difficult. The employment of the ordinary emmenagogues is very generally pernicious; and even the application of leeches to the feet, or to the vulva, will sometimes only aggravate the sufferings. Professor Mojon of Geneva assures us that he has used injections of carbonic acid gas per vaginam, with the most soothing effects. Like Rasori and Borda, he considers this gas as a powerfully depressing or contra-stimulant agent; and it was by reasoning from its known effects, as such, that he was led to try its effects as a local application to the womb in painful dysmenorrhœa. The gas is easily obtained by pouring diluted sulphuric acid on some pieces of chalk into a flask, (which ought to be provided with a double orifice,) like an inhaling apparatus;—a curved flexible tube is fitted on to one of these, and when the gas is freely disengaged, the extremity of the tube is to be introduced into the vagina, and the fumigation is to be continued for five or six minutes. This remedy may be used two or three times in the course of the day.

M. Mojon assures us that he has employed this mode of treatment in a great number of cases, and very generally with decided advantage. Not only was the pain almost always relieved for the time, but also the menstrual flow, in future, became more regular in its return, and more copious in its quantity.—*Bulletin General.*

---

### UTILITY OF THE FUMES OF CAMPHOR IN RHEUMATISM.

A labouring man, twenty-two years of age, had long suffered from attacks of flying rheumatism; but, as the pains were not severe, he neglected to use any remedial means.

Exposure to wet and cold brought on a smart attack of the disease in its acute form; and for this he had to undergo a vigorous antiphlogistic treatment by general and local bleedings, blistering, &c. The active symptoms were speedily subdued; but the patient continued to experience dull gnawing pains, increased by motion, sometimes in the loins, and at other times in the thighs and legs. Various means were used without much effect; and the physician was therefore induced to give a trial to the ingenious proposal of employing a vapour-bath of camphor-fumes, as recommended by M. Dupasquier in the Revue Medicale for 1826. The patient was made to sit on an open-seated stool, under which was placed a chafing dish. A plate of iron was then put on this dish, and, the patient being enveloped in a blanket, a small spoonful of powdered camphor was thrown, every five minutes, on the heated plate until about half an ounce had been used.—The vapour speedily induced a copious perspiration, and this was promoted by putting the patient into a warm-bed, and giving him copious diluent drinks. The first, and even the second, fumigation did not produce any very decided relief; but by the fourth day (for the treatment was repeated daily) the pains were greatly abated, and the freedom of motion much increased. Considerable debility followed the employment of this medication; but by appropriate means the strength of the patient was speedily restored, and he remained free from his rheumatic pains.—*Journal des Connoissances Med. Chirurg.*

---

### Spontaneous Hydrophobia.

A girl, 16 years of age, who had never menstruated, was seized with violent pains in the abdomen, attended with great constitutional disturbance.—Symptoms of dysphagia came on. All efforts to swallow were difficult and very painful. The mere sight however of fluids did not distress her. On the following day, a spasmodic stiffness affected the hands and arms. She was put into a warm bath, which at first she was afraid of entering. Towards evening a general convulsive agitation supervened; the tongue was protruded out of the mouth, and the larynx was forcibly drawn upwards, so as to threaten suffocation. Delirium, accompanied with lascivious expressions and behaviour, preceded death, which occurred at nine o'clock p.m. The dissection of the body was not permitted.

On reviewing the particulars of the preceding case, we regard it as one of the severe forms of Hysteria. It commenced with colic and terminated by nymphomania. No doubt the disease was connected with the changes in the female system, which precede menstruation. Leeches to the labia, and the internal use of camphor form the appropriate treatment in such cases.—*Schmidt's Jahrbucher.*

P. S. We quite agree with the Editor that the preceding case was one of the multiform varieties of Hysteria. It certainly has no title to be regarded as an instance of spontaneous Hydrophobia, as alleged by the reporter, who has occupied several pages of the German Journal in detailing particulars.—*Rev.*

---

### Veratria,—Remarks on the Employment of.

In recent numbers of the Journal de Pharmacie, and of the Bulletin General de Therapeutique, we observe two lengthened notices of Dr. Turnbull's work on Veratria, and the other analogous alkaloids, Delphinia, and Aconitine. The French Reviewers have not added anything to our stock of information on the preparation or medical use of these very potent remedies, nor have they reported any cases of their own, either to confirm or to impugn the accuracy of Dr. T's statements. We are the more surprised at this omission, as our continental brethren are certainly more disposed in general to give a trial to any novel medicines, more especially to such as are of a chemical or artificial nature, than British physicians usually are.

Since the date of Magendie's Formulary, there has not been—as far as we know—any published report in the French Journals of the therapeutic effects of Veratria and the other analogous salts, although the greater part of these medicines, hitherto in use, has been actually manufactured in Paris.

It is now nearly four years since we communicated in the pages of this review—(for July 1834)—some of the results of our own observations on the subject; and, as this communication was the first journalistic or critical notice of Dr. Turnbull's claims, we have felt some interest in ascertaining how far our own judgment has corresponded with the subsequent experience of other professional men. The last paragraph in our notice is thus worded:—"It is therefore our decided opinion that Veratria is a useful and very potent medicine in certain nervous affections, and that it deserves to be, and no doubt will become, an established member of the Materia Medica."

Our predictions have been realized. In the last edition of the London Pharmacopœia, we observe that Veratria and Aconitine are admitted, among the recognised preparations, by the College of Physicians.

It appears too, from various communications in the weekly medical journals, that medical men in different parts of the country have occasionally employed these alkaloids with marked benefit in cases of severe Neuralgia. In one of the recent numbers of the Lancet, we observe a short notice from that most witty of parsons—the Rev. Sydney Smith—of a case of most agonising *tic doloureux*, which was speedily relieved by the use of the Aconitine ointment—one grain to the drachm of lard. The complaint was of seven years' standing; and, at the date of the report, the patient had been totally free from pain for eight weeks.

The Doctor very justly adds—" She may relapse; but such an holiday in such a complaint is not to be forgotten." In our own practice, we have had more than one occasion to lament the *non-permanence of the relief*, from the Veratria treatment in severe Neuralgia. Still, it is a mighty object to procure temporary abatement of such distressing agony, as the poor sufferer has often to bear in this disease. We shall close these brief remarks by an extract from a communication by Dr. Young in the Quarterly Journal of the Calcutta Medical and Physical Society, for April 1837.

" Two drachms of Veratria were sent me by Mr. Snowdon of the Haymarket. I have used it in three cases of tic douloureux, and in two anomalous cases of nervous pain in the arm with marked success, fully equal to any thing stated by Dr. Turnbull in his work. It appears to me that any local pain in the nerves will be relieved, if not removed, by rubbing on the part, three or four times a day, a quarter of a drachm of an ointment composed of an ounce of lard and 15 grains of Veratria. The superintending surgeon of the Hyderabad forces has witnessed its extraordinary effects in a case of tic douloureux."

One hint to our readers—let them not forget the decided efficacy of many *old* remedies in even the worst cases of neuralgia. Perhaps on the whole none deserves our confidence better than *arsenic* administered internally.

---

SURGICAL APHORISMS. BY PROFESSOR WALTHER.

1. Tumors of the tongue are very rarely of a truly scirrhous nature. In the majority of cases, they may be dispersed by the application of leeches, and by frictions with the chloride of gold or of soda, in conjunction with appropriate constitutional treatment.

2. In enlargements of the prostate gland one of the most efficacious remedies is friction with the iodine ointment on the gland, by means of the finger introduced into the rectum. We have very frequently succeeded in curing the disease by this remedy alone.

3. Silver sounds are greatly preferable to common bougies, or elastic gum catheters in the treatment of strictures of the urethra. I have cured with a silver sound in the course of one week a stricture, which had baffled for several months all attempts with flexible instruments. The employment of caustic in the treatment of strictures is very rarely necessary, and a cure effected in this way is by no means more permanent than after the ordinary method.

4. Topical applications are of little or no service in active ophthalmia. Merely shading the eye is quite sufficient: occasionally indeed cold fomentations are useful.—*Schmidt's Jahrbucher*.

---

GENERAL REMARKS ON OPHTHALMIA.

From a memoir on Rheumatic Ophthalmia recently published by M. Sichel, we are induced to make a few extracts, as the author's descriptions are evidently drawn from an attentive examination of nature, and, although leaning perhaps somewhat too much to the doctrines of the school of Beer in Germany, suggest a useful comment on some of the errors of the Broussaian physicians. Of late years, it has been too much the fashion in France to decry the labours of the German ophthalmists, and to assert that the numerous species of Ophthalmia, described by them, may all be properly considered as only different degrees of one simple inflammatory disease. The influence of constitutional derangements in inducing ophthalmia has been too much neglected or denied; and hence the same line of treatment has been most perniciously recommended for the simple or catarrhal, for the scrofulous, the gouty, the rheumatic, and the syphilitic affections of the eye. Whatever be the form or type of the disease, these modern pathologists can discover, and will admit, nothing but mere vascular excitement or congestion; and if you talk to them of rheumatism, gout, or scrofula, they will ask you, "cet être que vous appelez rheumatisme, &c. l'avez vous vue?" But surely with as much show of reason, we might ask of them whether they had ever seen affinity or electricity. If experience shews to us that different cases, say of Conjunctivitis, differ, the one from the other, not only in the *visible* amount or extent of morbid action, but also in the mode of their origin, and of their progress, in their duration, and in their curability, ought we not fairly to conclude that these cases are in truth distinct affections? And if, pushing our enquiries a little further, we find that each of these cases coincides almost invariably with a certain state of the constitution, to which the names of rheumatic, gouty or scrofulous have been affixed, are we not bound to recognise the eye-affection as in some degree connected with, if not absolutely caused by, the general disorder? The justness of such a conclusion is surely confirmed, if the local complaint is remedied by the same means, which are known to be most efficacious against the disorder of the system. That such is the truth, will be admitted by all experienced ophthalmists, who have not blindly attached themselves to the Broussaian creed. We are ready to admit that many of the German oculists, with Beer at their head, have most unwisely, and most unnecessarily, multiplied their divisions of Ophthalmia; and, their perplexing minuteness of description and enumeration has perhaps contributed to the rise and growth of the opposite error, into which so many of the modern pathologists in France have fallen. It is quite as great a mistake in science to divide too minutely, as to generalise too vaguely. The Germans, armed, it would seem, with a sort of mental microscope, observe everything under a magnifying power; and, ingenious in distinguishing shades of difference, they multiply *genera* and *species* to excess, and end by having as many *types* as *individualities*. On the other hand the French have always been too fond of generalising, and, unwilling to exercise that 'religieuse patience' which is so necessary to the student of nature, they assemble together and classify things essentially very different from each other; the distinction of species and genera is forgotten; and they vainly attempt to simplify what is in truth multiform and complex.

M. Sichel, in his classification of the various sorts of Ophthalmia, has carefully avoided these two errors. He has neither multiplied their number unnecessarily, as Beer and his followers of the Vienna school have done; nor has he attempted to reduce them under one type, as the physiological oculists of France have of late years foolishly attempted. Guided by a calm discriminating judgment, and appealing constantly to the Book of Nature, he has been enabled, he thinks, to ascertain the causes of the different kinds of Ophthalmia, and he has satisfied himself that each kind has its seat in different structures of the eye, and is indicated by certain peculiar signs or symptoms, which the surgeon may by attention readily discover. The mucous membrane is, according to him, the part almost always affected in simple, and in catarrhal Ophthalmia; the fibrous structures in rheumatic Ophthalmia; and both of these structures usually suffer in the gouty or genuine arthritic Ophthalmia. This last species of ophthalmia is one of the most obstinate, which the oculist has to treat. Ophthalmia, when it occurs in strumous habits, is apt to be irregular in all its features. It has repeated periods of aggravation and of amendment; abating greatly for a few days at a time, and then returning with all its former severity. The disproportionate sensitiveness too of the eye to the apparent morbid changes is a characteristic feature of scrofulous ophthalmia. The tendency, which syphilitic ophthalmia has to affect the inner structures of the eye, is well known. In addition to these various constitutional causes of Ophthalmia, M. Sichel has repeatedly observed that abdominal congestions may be, if not the inducing, at least the predisposing cause of the disease. These various species of ophthalmia, to which we have now alluded, are occasionally complicated, and blended, as it were, the one with another in the same case. Thus the simple and catarrhal Ophthalmia may be grafted on the scrofulous; and this species again may co-exist with rheumatic or with gouty and syphilitic disease. Such cases require for their judicious treatment great tact and judgment on the part of the surgeon. Hence the importance of knowing thoroughly the constitutions of our patients, and the rise, progress, changes, and duration of their diseases, before we can form an accurate diagnosis or can adopt a rational mode of cure.

---

CASE OF XEROPHTHALMIA, (Ξηρος DRY) WITH REMARKS ON THIS RARE DISEASE. By M. VELPEAU.

French oculists have hitherto neglected to notice this affection of the eye—which consists in a morbid dryness of its surface—although Schmidt, Travers and Mackenzie have briefly described it.

The following is a well marked example, which occurred in the service of M. Velpeau at La Charité a few months ago.

A young man, of a robust although somewhat scrofulous constitution, had twelve months before his admission into the hospital suffered from inflammation of the right eye. An abscess formed at the time under the upper eyelid, and gave discharge to a quantity of pus from its inner surface. When this discharge ceased, the patient began to experience dull pains at the external part of the eye, also a gradual diminution of the lachrymal secretion and dimness of vision.

Various means were used, but without effect; and the surface of the cornea became quite dry, and the sight most indistinct.

When admitted into the hospital, it was observed that his right upper eyelid was somewhat inverted, and could not be elevated so much as the left one. The orifices of the meibomian glands and of the inferior lachrymal punctum were quite obliterated.

The caruncula lachrymalis was observed to be smaller than that of the other side, and imbedded in a triangular fold of the conjunctiva. This membrane presented a dull white colour and was "*entierement seche.*"

At both angles of the eye, it exhibited several vertical folds, which seemed to be more distinct and numerous in consequence of the efforts which the patient had made to separate the eyelids as much as possible.

When the eye-ball was drawn into the socket, the lower segment of the cornea seemed to be tied by one of these folds, as by the *membrana nictitans* in birds. The surface of the cornea itself was invested with a pulverulent pellicle, which was dry and unequally opaque. Through it, as through a cloud, the iris and pupil might be perceived. In short, the eye looked like the dry, dull and withered eye of a corpse, which had been exposed for a day or two to the action of the air; only with this exception, that it was not at all sunk in the socket. The patient had found that his sight was always clearer, if he moistened the surface of the cornea with water. When a solution of nitrate of silver (five grains to an ounce) was dropped into the eye, the patient experienced scarcely any pain or uneasiness.

The left eye was quite sound and had not suffered at all from any sympathetic influence with its fellow.

The treatment of Xerophthalmia or Xerosis has been hitherto utterly unsatisfactory. M. Villards has reported one case successfully treated by touching the conjunctiva around the edge of the cornea with the solid nitrate of silver; and M. Sanson has recommended the excision of the conjunctiva in the same place. M. Velpeau tried, in the present case, the solution of the lunar caustic and also the insufflation of calomel, but without having obtained any decided benefit.

*Remarks.* The rare disease of Xerophthalmia has been strangely confounded by some authors with some kinds of opacity of the cornea. The two affections are altogether different from each other. In the former, the lamellar tissue of the cornea appears to be sound; and it is only the conjunctival epithelium investing it, which becomes dry, thickened, opaque, and, as it were, "epidermifie." It has a slatey hue, is pulverulent, and is of a bedimmed transparency which impairs, without abolishing, the visual functions. On the other hand, leucoma, albugo, &c. are diseases of the cornea itself; the opacity in them is much more decided, and, if situated over the axis of vision, it is attended with an almost total blindness. With respect to the causes of Xerophthalmia, we have no satisfactory information. Schmidt, Travers, and others attribute the disease to an obliteration, more or less complete, of the excretory ducts of the lachrymal gland. But this assertion is quite gratuitous. These alleged ducts have never been shewn; no necroscopic examination of a "xerosed" eye has ever been made; and lastly M. Magendie has found that the extirpation of the lachrymal gland in the lower animals is not followed by desiccation of the cornea—its transparency and humidity being probably maintained by the mucous secretion from the papillæ of the conjunctiva, from the Meibomian gland, and from the caruncula lachrymalis. May not the disease be owing to an affection of the ophthalmic branch of the trigeminus nerve—the nerve which Magendie believes to be "l'agent presque exclusivement conducteur de la sensibilité dans les organes des sens?" The diminution of vision and of the sensibility of the conjunctiva, the imperfect and embarrassed movements of the eyelids, and the cessation of the lachrymal secretion—all these phenomena may perhaps be explained on this hypothesis. Whether the formation of the abscess in the upper eyelid had any influence in inducing the Xerophthalmia in the preceding case, is not easily determined. If it had, some will allege that the lachrymal gland itself was implicated, and others perhaps may suppose that the frontal nerve suffered from its contiguity to the abscess, and that the other branches of the ophthalmic became affected from sympathy.

On the whole we are rather inclined to attribute the origin of this rare disease to the effects of chronic catarrho-strumous conjunctivitis, than to any other morbid state with which we are acquainted.—*Gazette Medicale.*

---

### On a New Means of Diagnosis between Amaurosis and Cataract. By M. Sanson.

If a light be presented before an amaurotic eye—the pupil of which is either naturally or artificially dilated—three distinct images of the flame may be invariably observed. Of these three images two are upright, and one is *reversed:* they are situated, the one behind the other, in the following order. The anterior one, which is also most distinct, is one of the former or *upright* images. The posterior or deepest, which is the least distinct, is also one of the upright images. The intermediate image is the *reversed* one.

This last or reversed image is paler than the first, but brighter than the second, upright one; and it also differs in this circumstance, that, when the light is moved to either side or round the eye, it is separated from the other two images so as always to occupy the opposite side, while they (the upright ones) are seen to follow the position of the light, moving to the right or left, upwards or downwards, according as the candle is moved in any of these directions.

If the candle be held opposite to the axis of the eye, all the three images are situated one behind the other—the two posterior ones being, as a matter of course, masked and obscured by the anterior one. But if it be held to a—say the right—side, then the reversed image will be seen in the opposite or left angle of the eye, while the upright ones are seen at its right angle.

If it be moved around the eye, the upright images follow it together, while the reversed image, although describing the circle in the same direction, is always at the opposite end of the eye's diameter.

The unpractised observer may experience some difficulty in observing these phenomena.

The patient should be placed in a dark chamber; and let us suppose that the candle is held at the external angle of the eye: the anterior upright image, which is large and brilliant, will be observed at the outer and upper part of the pupil. If we now look very attentively into the bottom of the eye, the reversed image will be seen at about one line's breadth from the preceding upright image and at the meeting of the lower with the middle third of the diameter of the pupil—the right extremity of which (the diameter) is occupied with the anterior upright image.

If the surgeon does not detect these phenomena at first, he has only to move the light upwards and downwards, once or twice, fixing his look steadily on the pupil, and he cannot fail to observe that one image rises and the other descends.

As to the posterior or deep-seated upright image, it is always very difficult to perceive it, in consequence of its paleness, and of the intervention of the other upright one—of which it looks like the shadow.

M. Sanson assures the surgeon that, when once they have detected the very images, they will always readily perceive them afterwards, *provided there be no obscurity or opacity of the lens.*

Whenever a cataract exists, no matter what may be the stage or progress of the disease, none of the images, described above, are ever perceptible.

Some time ago (says M. Sanson), a patient was sent to me from a great distance to be relieved by operation from a cataract: *the three images were perceived; the patient was affected with glaucoma.*

A few days ago I was desired to visit a patient, who had been pronounced by several medical men in the metropolis to be affected with cataract: I perceived the three images and declared the case to be one of amaurosis.

You (his hearers) have lately seen a woman, whose sight was entirely lost. She had been sent to my care as an amaurotic patient. There was no opacity visible in the field of the pupil; but two of the images were absent. I gave it as my opinion that she had two cataracts; and the accuracy of this diagnosis has been subsequently confirmed.

The preceding remarks were made by M. Sanson, one of the surgeons of the Hôtel Dieu in Paris, in his course of lectures on ophthalmology during last year. He had first noticed the phenomena, described above, about twelve months previously; and he had availed himself of his ample opportunities in the hospital during this period to test the accuracy of his opinion. He assures us that his experience has quite satisfied him of its truth.—*l'Experience; Journal de Medicine et Chirurgie.*

---

### On the Treatment of Amaurosis by Cauterization of the Cornea.

M. Lisfranc, the eminent surgeon of La Pitié Hospital, has been in the habit for some years past of treating numerous cases of Amaurosis by the application of nitrate of silver to the cornea. In his opinion, it is especially useful in those cases, where it is desirable to excite the branches of the fifth pair of nerves, as well as the general vascular and nervous apparatus of the eyes. The objection, that this treatment is apt to induce various degrees of ophthalmic inflammation, is easily avoided by applying the caustic very gently at first, so that the surface of the cornea becomes, as it were, accustomed to the irritation gradually. It is not necessary ever to apply the caustic on the middle of the cornea; all that is requisite is to touch several points in its circumference, so as to induce slight pain and vascular injection.

One speedy effect of this mode of treatment is to restore the contractility of the iris, and also to excite a general *erethism*, so to speak, of all the tissues of the eye. This is followed by a copious secretion of tears, and of the nasal mucus, and by smart pain in the forehead and cheeks; the capillary vessels are injected, the pupil becomes contracted, and the retina more sensitive to the impression of light. We might adduce numerous cases to shew the beneficial effects of this mode of treatment in amaurotic blindness.

The following one is taken from M. Lisfranc's Clinique at La Pitié.

An optician, 38 years of age, plethoric and robust, was admitted on the 20th of October.

For the preceding five months, he had found his vision becoming less and less distinct; objects appearing to him surrounded with a mist, and sometimes with luminous circles. He had been treated with blisters applied to the forehead; but, having derived no benefit, he determined to consult M. Lisfranc.

On his admission into the hospital his bodily health appeared to be quite good; but many of his actions indicated a disposition to mental alienation. Upon inquiring into the history of his former life, it was discovered that some years past his conduct had on many occasions been most irregular, if not absolutely insane.

The eye exhibited no signs of structural disturbance; but the pupil was largely dilated, and did not contract on exposure to light. Although able to distinguish light from darkness, he was quite incapable of guiding himself.

On the 25th, M. Lisfranc applied the nitrate of silver to the lower part of the cornea, along the extent of two lines or so. On the following day the conjunctivæ were found to be reddened, the eyes were brilliant; there was a copious secretion of tears; and the patient complained of severe head-ache: his pulse was frequent and full.

He was accordingly bled; and the eye was ordered to be bathed with an emollient fomentation. This treatment was continued for four days, when the inflammatory symptoms were found to be nearly gone. The power of distinguishing objects gradually returned. He remained in the hospital for six weeks; and by that time his vision had very greatly improved.—*Bulletin General de Therapeutique.*

*Remarks.*—The preceding case is very absurdly adduced as a proof of the utility of the *cauterization* treatment in amaurosis.

Judging from the report the cure, if such it was, was clearly attributable to the bleeding and other antiphlogistic measures, and not certainly to the application of the nitrate of silver to the cornea.

We do not mean to condemn this treatment—far from it; it is certainly very useful in many cases of amaurotic blindness. We only deprecate such injudicious advocacy of it, as the adducing of such cases as that now extracted from the Bulletin.

Unless judgment is exercised in the selection of cases, no mode of treatment in amaurosis, or in any other disease, can possibly be expected to be of extensive benefit.

The French journalists appear to give the entire credit of the use of nitrate of silver in amaurosis to M. Lisfranc.—This is certainly not quite fair: they surely must know how largely it has been used, and how ably it has been recommended, for many years past, by our countryman Mr. Guthrie.

---

### Extirpation of the Uterus.

MM. Capuron and Lisfranc lately read to the Academy their report on the following case. Professor Laserre was consulted by a middle-aged woman for an inversion and prolapsus of the womb, which had been induced by the violent traction employed by a midwife at her last accouchement, two years before. From that period, she had suffered repeatedly from alarming uterine hæmorrage.

The tumor was of a pyriform shape, irregular and knobby on its surface, and the narrowest part or pedicle was girt with a hard ring (bourrelet) in the situation of the uterine orifice. Finding that it was quite impracticable to replace the uterus, M. Laserre determined to extirpate it, by means of a ligature passed round its pedicle, and tightened as firmly as the patient could bear. The acute pain induced by the ligature was calmed by opiates. 'Apres quelque temps' a second ligature was applied more tightly; but this operation caused such severe suffering, that it was found necessary to remove the string. The attempt was subsequently made several times; but the same alarming symptoms always coming on, the surgeon was obliged to resort to another practice. Drawing the tumor out, as far as he could, and finding that part only of the pedicle had been destroyed by the ligature, he passed another ligature tightly round it, and then removed the mass with one stroke of the knife. Peritonitis supervened; but a rigorous antiphlogistic practice speedily dissipated all the unpleasant symptoms. At the end of the fifth day 'l'etat de la malade etait satisfaisant.' On the following week, a painful swelling of the left thigh and leg came on; but this also gradually subsided, and in the course of five weeks from the date of the operation, the cure was complete. When the case was laid before the Academy last June, a year had elapsed since the period of cure. During that time, there had been no appearance of catamenia, and not even the 'prodromes' of the discharge had been experienced; whence M. Laserre concludes that this secretion must proceed altogether from the uterus and not from the vagina. The patient has been able, we are informed, 'se livrer au coit comme avant, et elle y approuve la meme jouissance et les memes sensations.'

M. Capuron, in his report to the Academy on the preceding case, expressed his conviction that the uterus had been really extirpated; but adds, that it would have been more satisfactory if the details of the examination and of the characters of the prolapsed tumor had been more minutely and explicitly given. With respect to the absence of the catamenia, M. Laserre has been rather precipitate in his conclusion that they will never return in future; and he appears not to be aware that in several of the recorded cases of extirpated uterus, the menstrual discharge has been renewed, after having been absent for several years.

M. Nacquart directed the attention of the members to a coloured engraving, intended to represent the oozing of the menstrual discharge from the mucous surface of the vagina, in a thesis published by Osiander.—*Archives Generales.*

---

RADICAL CURE OF PROLAPSUS UTERI.

M. Velpeau recently performed the following operation in an old case of prolapsus of the womb, complicated with a cystocele.

Pinching together the mucous coat of the vagina, he cut away three slips of it, one from the anterior part, and the other two from the sides of the canal. Each of the slips was nearly an inch wide, and two inches and a half long. M. Velpeau had previously inserted the ligatures, so that there was no difficulty experienced in bringing the edges of the wound together.

No accident followed the operation, and cicatrisation took place by the first intention. Two months had elapsed, at the date of the communication to the Academy; and there was therefore every reason to believe that the relief would be permanent.

M. Berard, surgeon of the Hospital St. Antoine, stated that he had performed the same operation in three cases of prolapsus uteri, and in two of them a complete cure was obtained.

---

TREATMENT OF BURNS, BY M. VELPEAU, WITH DIACHYLON PLASTER.

The ever active surgeon of the La Charité recommends the employment of strips of diachylon plaister to burned surfaces, in preference to the ordinary applications, such as cold water, solution of the chlorides, Carron oil, &c. The advantage which it possesses over these means, according to M. V., is its being well adapted for all the various stages or degrees of the injury — from the simple irritation of the skin, caused by scalding, to the complete destruction and mortification of it and the subjacent cellular tissue. He recommends that the strips of the plaister be renewed every two or three days, and assures us that burns 'du premier degré guerissent immediatement;' those of the second degree in four or six days; those of the third degree in from eight to fifteen days; and those of the fourth degree in from fifteen to thirty days.

When the injury is attended with a destruction of the parts, and is therefore to be cured by ulceration and granulation, the process of cicatrisation goes on under the plaister in several points at the same time, and not merely 'de proche en proche,' or from the circumference to the centre; as is ordinarily the case under the common dressings.

---

### Injections of Iodine in Hydrocele.

M. Velpeau remarks that perhaps there is no surgical disease, in which less change of description or of treatment has been made use of for the last half-century than Hydrocele. Authors, for many a year now, have satisfied themselves with merely repeating in different words the statements of their predecessors. With respect to the treatment of hydrocele in France, the mode by puncturing and injecting the sac is almost universally adopted. But independently of the occasional failure of this method, it is liable to not a few incoveniences. Let us briefly examine the merits of the other methods, by which hydrocele has been treated.

*Acupuncture.*—This simple operation will sometimes suffice to effect a permanent cure. M. Velpeau alludes to a case, where the disease, of three years' standing, was effectually and speedily dissipated by the accidental insertion of a long needle into the scrotum.

Dr. Monro mentions a case in his practice, where he cured, in the space of six days, a hydrocele by introducing a long needle into the sac, and leaving it there to act as a seton.

The numerous experiments, which I (Velpeau) have performed on arteries and veins, with acupuncturation has quite satisfied me of the admirably curative effects of this remedy in causing inflammation and obliteration of serous cavities. In 1836 I tried this method in two cases of hydrocele; but in both it failed of success. In two other cases, I treated the swelling with leaving in the sac a small seton for two or three days. Upon removing the threads, suppuration ensued, and a cure was ultimately effected; although more slowly than after the method by the port-wine injection. The following case, interesting in several points of view, affords a proof of this remark.

A man, 27 years of age, of a healthy constitution, was admitted into the Charité Hospital, in consequence of a double hydrocele, which had existed for several years. M. Velpeau proposed to try the radical cure with the vinous injection on the left sac, and the palliative method on the right one. While the injection was going on, the patient struggled and pushed the canula away from him. This was no doubt the cause of a very little of the wine having escaped into the cellular substance of the scrotum. For several days, however, there was no tumefaction or pain. About the 4th or 5th day however after the operation, the patient began to complain of shooting pains through the left testicle, which had now become somewhat swollen and tender. Some of the inguinal glands also exhibited a tendency to enlargment. The swelling of the scrotum gradually increased, and an erysipelatous redness was diffused over the surrounding integuments, which exhibited a tense shining aspect.

The puncture, which had never healed, enlarged, and displayed at its base a semi-fluid yellow coloured substance. The parts immediately surrounding the puncture were rather soft and crepitating on pressure.

On the 10th day after the operation, M. Velpeau made an incision, an inch and a half long, upon the tumor, and gave discharge to some yellowish pus, mixed with bubbles of air. At the bottom of the incision there was observed a dark, almost black, eschar, which was pulled away with the forceps. For several days, the suppuration was very scanty, and the wound exhibited an unhealthy appearance.—Towards the end of the 4th week, the patient left the hospital to return to his work. At this period, the left sac was quite empty, but a re-accumulation had taken place in the right one.

He had not however been away more than 10 days, when he returned for admission into the hospital. The incision was still open; and now it was discovered that fluid had collected in the left, as well as in the right, tunica vaginalis. A few days after this date, M. Velpeau passed two small setons through both hydroceles. On the second evening, the patient began to suffer from colicy pains, hiccup and vomiting, and to become generally feverish and uneasy.

On the following day the constitutional disturbance continued; and the scrotum, especially on the left side, was now extremely painful. The spermatic cords also were hard and tender to the touch. Both setons accordingly were withdrawn, and the patient was bled from the arm.

For the next fortnight, the patient was in a very unpleasant state, and the local symptoms continued to be very severe. There was still a considerable quantity of fluid in both sacs. At the end of another week, the poor fellow again left the hospital; but it seems that he found his condition at home so uncomfortable, that he was glad to be re-admitted in the course of a few days. The report states that, at this time, he suffered much pain in the right groin; that the right cord was hard, swollen, and very tender on pressure, and that all the right side of the scrotum was as hard and unyielding to the touch as a piece of wood. To add to his sufferings, his gums were tender from the mercurial frictions, which had been employed.

He was ordered an alum gargle; to have 25 leeches to the right groin, poultices and saturnine wash to the scrotum.

For the following week, he was put upon the use of large doses of the tartrate of antimony, and what with being vomited, purged, and otherwise physicked, the "bien infortuné" was glad to be discharged at the month's end! His state at this time is thus narrated in the report:—"Il s'est fait un notable degorgement dans la tumeur; il n'y a point de rougeur, mais il y a toujours de l'induration. L'hydrocele droite, qui n'a pas été guerie en entier, a diminueé; la gauche est completement fermée."

In conclusion, adds M. Velpeau, I am of opinion that acupuncturation, whether with or without the use of the seton, is a process to be definitively rejected from the treatment of hydrocele.*

The following are a few cases, in which M. Velpeau has used an iodine injection in the cure of hydrocele.†

A man, 61 years of age, fell down and bruised his scrotum against the edge of a saddle, which was lying on the ground. A week afterwards, the swelling of the scrotum presented all the characters of a hydrocele. M. Velpeau punctured it with a trocar, and discharged half a wine-glassful of yellow-coloured serum. The fluid re-accumulated in the course of a few weeks, and it was again let out by puncture, and then

* Within the last twelve months, several of the contributors to the Medical Gazette have published cases, in which simple acupuncturation sufficed to cure hydrocele. The plan may answer in young patients, but not generally in the aged.—*Rev.*

† The injection was usually composed of one drachm of tincture of iodine to an ounce of water.—(Query. Is the French tincture of iodine as strong, as what is used in this country?)

a weak solution of iodine was immediately afterwards injected into the sac. Judging from the report, it appears that very little inflammation and swelling followed. The cure nevertheless was complete.

In the *second case*, the hydrocele was very recent and inconsiderable in size. It seemed to be the result of a hernia humoralis, which had been brought on by the retrocession of a gonorrhœal discharge. The fluid was simply drawn off by puncture at first; but, on the second operation, M. Velpeau injected a weak solution of iodine, immediately after discharging the fluid. The cure was very speedy, and remained permanent.

M. Velpeau has used the iodine injection in more than *twenty cases*, and in all with perfect success. In two of these successful cases, the vinous injection had previously failed; and in the other two there was encysted hydrocele of the cord. We shall give some short particulars of two or three of his cases.

The first we shall mention is that of a youth, nineteen years of age, who had been affected with hydrocele for a few months. A short time before he applied to M. Velpeau, a surgeon had discharged the fluid and had injected some diluted port wine. But this operation seems to have failed, and M. Velpeau determined to try the iodine solution. About a wine-glassful of citrine-coloured serosity was withdrawn, and then an ounce and a half of the solution (containing two drachms of the tincture) were injected.

After remaining in the sac for some time, one half of it was allowed to flow out, the other half remaining behind.

Immediately after the operation, the patient walked off to his home. (We must confess our surprise that any patient was able to do this. The injection of hydrocele with a stimulating fluid is usually a very painful operation, and most patients are glad to get to bed.—*Rev.*) Six days afterwards he came to M. Velpeau's consultation. He had not kept his bed, nor made any change in his diet. In another eight days he was pronounced cured.

A medical student, 22 years of age, had been affected with hydrocele for five years, when he applied to M. Velpeau.

The swelling was as large as the head of a six-month's fœtus. Eight ounces of serosity were withdrawn by puncture, and immediately afterwards a mixture of six drachms of tincture of iodine with four ounces of water was injected into the sac. The whole of this quantity was not injected at once; about two ounces were first used, and when this quantity was permitted to escape, the rest was introduced. The patient experienced no pain either in the scrotum, or in the back! On the following day the scrotum became swollen, tender and painful. The progress of the case was quite satisfactory, and the cure was pronounced at the end of the second week.

In another case, in which two drachms of the tincture mixed with two ounces of water were injected, the patient experienced so little inconvenience that he rose to dinner a few hours after the operation, and drove out that evening! His sleep and appetite remained as good as ever. A slight degree of swelling and pain succeeded, but these symptoms speedily gave way, without requiring any confinement on the part of the patient. The cure was complete by the twelfth day.

A similar mode of treatment has been followed by M. Velpeau in numerous other cases with perfect success. From two to six drachms of the tincture mixed with water were the usual quantity injected.

Part of the injection was sometimes allowed to remain in the sac; and so little inconvenience did this produce, that the patients walked home after the operation, and did not require to confine themselves to bed at all. It is to be remarked that, in not a few of the cases, the collection of fluid in the vaginal sac was very small: as we find it mentioned in the reports, that not more than a wine-glassful was drawn off by the canula.

M. Velpeau is of opinion that the iodine injection is preferable to the port-wine one, for several reasons. It has been most unequivocally proved, by the results of several of the cases alluded to above, that it is a fluid capable of being absorbed.

From this we may reasonably infer that its infiltration into the cellular substance will not be attended with so much serious mischief, as when the vinous mixture becomes extravasated. It causes also decidedly less pain than the wine; and indeed in most cases the pain is so inconsiderable, that the patient is not obliged to go to or to keep his bed. It has succeeded, when the vinous injection has failed.

From the results of his experiments, M. Velpeau is led to anticipate that the iodine injection will eventually be substituted for the port-wine one. He admits that as yet he has not quite satisfied himself, what is exactly the best proportion of the tincture to be used; whether any portion of the fluid should be allowed to remain in the sac; whether confinement to bed after the operation expedites the cure, &c. These are points to be determined by future researches.

In one case, in which the iodine mixture was used, the patient died about six weeks after the operation, in consequence of having had one of his limbs amputated. On examining the scrotum, it was found that the tunica vaginalis was about two lines thick, and adhered very intimately to the testicle, so that any relapse of the effusion was impossible.—*Archives Generales.*

---

### Treatment of Strictures of the Urethra by Bougies coated with Calcined Alum.

M. Jobert, one of the surgeons of the Hôpital St. Louis in Paris, presented lately to the Institute a memoir on this subject. We shall extract two or three of the cases reported, and leave his reasonings alone. A fact is worth a ship-load of ideas. The manner, in which he prepares his bougies, is this. He smears the one about to be used with oil, and then rolls it in calcined alum finely pulverized. Sometimes he has made use of a soft ointment, with which the alum has been previously well blended.

A middle-aged man caught a severe gonorrhœa in 1828. It was followed by a complete obstruction of the urethra, which had given way at one part, and permitted an extravasation of urine into the cellular tissue of the perineum, scrotum, &c. He recovered from the painful effects of this accident, but the difficulty of passing urine still remained. On the 19th of April, 1835, he was admitted into the Hôpital St. Louis: two strictures were discovered. Several attempts to dilate the urethra were made, but in vain; and M. Jobert, therefore, tried to pass one well smeared with the oil and alum. Considerable pain was produced, and several drops of blood flowed out; but in the evening the patient confessed that he had passed his urine with greater ease than he had done for seven years before. Next evening another bougie, prepared in the same manner, was passed, and allowed to remain in the canal for some hours. The report ceases very abruptly here!

The *second case* was of a longer duration and of greater difficulty. The patient had suffered from repeated gonorrhœa, and attacks of ischuria. When he entered the hospital, the smallest-sized bougie could not be passed more than four inches into the urethra. After being well smeared with the oil and powdered alum, it was re-passed and secured in its place. On each succeeding day a bougie of larger size, and similarly medicated, was passed along and kept in the canal for several hours. Considerable smarting was caused, and there was an abundant flow of mucus from the urethra. (This mode of treatment was continued for a week or so, and we are told, that in that time the disease was cured. But such vague statements will not satisfy British surgeons, however worthy to be submitted to the French Institute.—*Rev.*)

The *last* reported *case* deserves more particular notice. A man, 37 years of age, had contracted gonorrhœa in 1821: it was very obstinate, but after four or five months' duration it gradually ceased. In 1830 he was again diseased; the dysuria was very severe, and, after the discharge was stopped, the passage was found to have become strictured. The use of bougies was repeatedly tried, but always with only temporary relief; and so troublesome was the case, that he had been advised by one of the surgeons in Paris to submit to the use of caustic bougies. He went into the Hôpital St. Louis in Feb. 1836. M. Jobert attempted to introduce a small bougie into the bladder, but it could not be passed beyond the bulb; and, as the discharge of the urine was always in drops, and never in an uninterrupted flow, it was suspected that the constriction of the urethra was very great. During the next fortnight various attempts were made to overcome the stricture; but all proved ineffectual. M. Jobert then introduced a bougie oiled and "saupoudrée" with calcined alum, and pressed it with considerable force against the obstruction. This operation was repeated daily for the next six days, and it was then found practicable to pass the bougie fairly into the bladder. —*La Lançette Française.*

---

### Spontaneous Cure of an Aneurism of the Iliac Artery.

A sailor, 34 years of age, after lifting a heavy weight, found a small swelling in his right groin, which was accompanied with slight pain and stiffness of the limb on attempting to move it. The surgeon, to whom he first shewed it, had quite mistaken its real nature; for he had prescribed mercurial frictions, leeches, &c. He was left at the hospital in Smyrna, in consequence of his inability to perform the duties on board his ship; but there too the surgeon seems not to have been aware of his disease, although the tumor by this time had become considerably larger, and pulsated strongly: the skin over it too was discoloured, and the whole limb stiff and somewhat œdematous. Fortunately the surgeon of the French frigate Galatea happened to see the case, and at once resolved to carry the poor fellow back to France.

The following was the state of the swelling at this period. It was situated a little below the fold of the groin, extending down rather more on the front of the thigh than upwards on the abdomen; its base was diffused, but towards its summit it was more circumscribed. Pressure caused but little pain, and there was no greater heat in the part than elsewhere.

With attention, a distinct pulsation, isochronous with the action of the heart, might be felt. Compression above the tumor produced but little change upon it; but compression below it at once caused it to become larger and more prominent. The limb was œdematous and incapable of movement.

He was two months on board the frigate; and during this time the state of the swelling, and of the limb generally, improved considerably, in consequence of the depletory treatment employed, and the rest, which was diligently attended to.

When transferred to the hospital at Toulon, a minute examination of the limb was made. It was found to be nearly twice as large, at the upper part of the thigh, as the opposite one. The base of the tumor extended from the superior anterior spine of the os ilii forwards to the linea alba, and downwards to the upper third of the thigh, measuring in circumference twenty-two inches in all. The whole surface was firm and unyielding, except at its summit, where it was slightly softer and more compressible. The very central point gave to the finger a sense of fluctuation; and there the skin was purplish, and appeared to be so thin as if it threatened to burst. The pulsation, which was indistinct to the hand, could be more readily perceived, when the ear was applied to the swelling. The ligament of Poupart was raised up, and divided the swelling into an upper and a lower portion. The whole limb was excessively œdematous, and quite incapable of voluntary motion.

Such was the condition of this large aneurism six months after its commencement. It was the unanimous opinion of all the surgeons that not only was the crural and also external iliac portion of the artery diseased, but the common iliac was involved at the same time. This opinion was founded on the circumstance of a firm unyielding cord being traced from the upper part of the swelling towards the umbilicus. (L'abdomen exploré avec attention donne, le long le trajet de l'iliaque externe, la sensation d'un cordon dur, volumineux, se prolongeant jusqu' au dela du promontoire du sacrum vers l'ombilic, où il semble se terminer.)

The firm unyielding character of the swelling, and the indistinctness of the pulsatory movements, suggested the idea that it was partly filled with coagula. The œdema, coldness, and numbness of the limb, were no doubt attributable to the compression of the crural vein and nerves.

The question to be now determined was, whether an operation should be attempted, (and if any operation, it must have been that of tying the aorta)—or whether the case should be left to nature, and means be used to promote the coagulation of the blood in the sac. The latter plan was most fortunately adopted; and it was resolved to give a fair trial to the treatment of Valsalva. It was commenced on the 18th of February.

The patient was put on a light farinaceous diet, and a bladder of ice was retained constantly on the tumor.

It is quite unnecessary to detail the particulars of each report. Suffice it to say that the size of the swelling began very sensibly to diminish: the œdema of the limb and the numbness also became less and less.

The treatment appears to have been steadily persevered in till the beginning of November—eight months—at which time the use of compression, by means of a rolled bandage applied along the whole extent of the limb, was commenced.

By the beginning of the new year, 1835, the limb had nearly recovered its healthy dimensions, and a considerable degree of pliancy and power of movement. No pulse, however, could be discovered either in the popliteal or in any of the plantar arteries. Soon afterwards the patient tried to move about on crutches; but, the attempt bringing on a swelling in the limb and pain in the groin, it was immediately discontinued.

It would seem that the patient remained in the hospital for other twelve months, and that the application of ice to the tumor was continued for most of that time.

A feeble pulsation began to be detected in the lower third of the femoral and also in the tibial arteries.

The patient was at length discharged perfectly and most satisfactorily cured. —*Gazette Medicale de Paris.*

---

PERFORATION OF THE VERTEBRAL COLUMN BY AN ANEURISM OF THE AORTA, NOT SUSPECTED DURING LIFE.—SYMPTOMS OF GENERAL TETANUS.

A porter, 38 years of age, and of an athletic constitution, was admitted into the Hospital *Santa Maria della Scala* at Sienna, in consequence of a suspected affection of the spinal cord. For eight months he had been subject to spasms of the limbs—the spasms occasionally alternating with a paralytic weakness of these parts. During his residence in the hospital, he had repeated attacks of spasmodic contractions in the muscles of a part of the trunk, or of the extremities; and those attacks were so severe as to exhibit all the varieties of tetanus, known by the names of *Opisthotonos, Emprosthotonos,* and *Pleurosthotonos.* His pulse however, was never irregular or febrile; and his mental faculties were all the while quite unaffected. His appetite was voracious, almost insatiable; and occasionally he suffered from insupportable burning pains in the bowels. He died suddenly during a tetanic attack.

His disease was suspected to consist in a chronic inflammation of the spinal marrow. On dissection, an aneurismal tumor, of the size of a hen's egg, was found upon the posterior surface of the arch of the aorta. The bodies of the third and fourth dorsal vertebræ had been quite absorbed, so that the swelling lay in the spinal canal. The cord and its membranes did not however exhibit any traces of morbid change.—*Fragmens d'un Voyage Medicale en Italie, dans le Bull. Med. Belge.*

